# Supplementary material for: Recovery priorities in degenerative cervical myelopathy: a cross-sectional survey of an international, online community of patients
Source: BMJ Open. 2019 Oct 10;9(10):e031486. doi: 10.1136/bmjopen-2019-031486 (PMC6797315; doi:10.1136/bmjopen-2019-031486)
Supplement: Supplementary data [file bmjopen-2019-031486supp001.pdf]

Supplementary Data 1: Results from the missing data analysis. Patients completing the survey in full, were more likely to have undergone surgery than those who did not (*p* = 0.04). Data is presented as mean +/- standard deviation, unless specified as a percentage. Numbers within brackets indicate data points for the respective variable with incomplete data.

	INCOMPLETE SURVEYS (N<178)	COMPLETED SURVEYS (N=481)	P
**%MALE**	16.3% (7/43)	29.1% (140/481)	.076
**AGE**	55.1 +/-10.9 (43)	53.6 +/- 9.8	.344
**%SURGERY**	35.9% (46/128)	45.9% (221/481)	.043
**LENGTH OF SYMPTOMS (YRS)**			.304
**0-1**	18.0% (23)	15.0% (72)	
**1-3**	21.9% (28)	29.1% (140)	
**3-10**	35.2% (45)	37.6% (181)	
**10-25**	18.8% (24)	15.4% (74)	
**25+**	6.3% (8)	2.9% (14)	
**LIMB PAIN VAS**	3.6 +/- 1.9 (17)	3.1 +/- 2.6	.854
**MJOA**	12.2 +/- 3.2 (47)	11.9 +/- 3.0	.364
