# Supplementary material for: Recovery priorities in degenerative cervical myelopathy: a cross-sectional survey of an international, online community of patients
Source: BMJ Open. 2019 Oct 10;9(10):e031486. doi: 10.1136/bmjopen-2019-031486 (PMC6797315; doi:10.1136/bmjopen-2019-031486)
Supplement: Supplementary data [file bmjopen-2019-031486supp002.pdf]

Supplementary Data 2: Summary of group differences between investigated variables. Highlighted cells represent significant differences in the proportion of respondents per group. Further analysis revealed that differences followed a logical course: patients who had had symptoms for longer or had undergone surgery were more likely to have severe disease. In addition, patients with more severe disease were more likely to have higher limb pain scores.

	N	Age (±SD)		Male (%)		Undergone Surgery (%)		Length of Symptoms <3yrs (%)		mJOA Upper Limb Function <3		mJOA Lower Limb Function <4		mJOA Upper Limb Sensation <2		mJOA Bladder Function <2		Mean VAS Limb Pain <3	
Gender, Male	140	55.8	10.3			64	46%	71	51%	20	14%	51	36%	50	36%	30	21%	88	63%
Gender, Female	341	52.8	9.5			157	46%	141	41%	38	11%	107	31%	143	42%	75	22%	205	60%
Undergone Surgery	221	53.1	8.7	64	29%			83	38%	31	14%	90	41%	101	46%	53	24%	135	61%
Not Undergone Surgery	260	54	10.7	74	28%			129	50%	27	10%	68	26%	92	35%	53	20%	158	61%
mJOA Upper Limb Function <3	58	56.7	9.3	19	33%	31	53%	13	22%			46	79%	52	90%	29	50%	21	36%
mJOA Upper Limb Function >3	423	53.2	9.8	119	28%	190	45%	199	47%			112	26%	141	33%	76	18%	272	64%
mJOA Lower Limb Function <4	158	55.4	10.1	50	32%	90	57%	51	32%	46	29%			91	58%	61	39%	85	54%
mJOA Lower Limb Function >4	323	52.7	9.5	88	27%	131	41%	161	50%	12	4%			102	32%	44	14%	208	64%
mJOA Upper Limb Sensation <2	193	53.7	9.1	48	25%	101	52%	78	40%	52	27%	91	47%			67	35%	86	45%
mJOA Upper Limb Sensation >2	288	53.3	10.3	90	31%	120	42%	134	47%	6	2%	67	23%			38	13%	207	72%
mJOA Bladder Function <2	105	54	9.6	29	28%	53	50%	40	38%	29	28%	61	58%	67	64%			58	55%
mJOA Bladder Function >2	376	53.5	9.9	109	29%	168	45%	172	46%	29	8%	97	26%	126	34%			235	63%
Length of Symptoms <3 years	212	52.8	10.8	71	33%	83	39%			13	6%	51	24%	78	37%	40	19%	136	64%
Length of Symptoms >3 years	269	54.2	8.9	67	25%	138	51%			45	17%	107	40%	115	43%	65	24%	157	58%
Best Limb Pain VAS <3	293	53.3	11	88	30%	135	46%	136	46%	21	7%	85	29%	86	29%	58	20%		
Best Limb Pain VAS >3	188	53.6	8.6	52	28%	86	46%	76	40%	37	20%	73	39%	107	57%	47	25%		
