# Supplementary material for: Recovery priorities in degenerative cervical myelopathy: a cross-sectional survey of an international, online community of patients
Source: BMJ Open. 2019 Oct 10;9(10):e031486. doi: 10.1136/bmjopen-2019-031486 (PMC6797315; doi:10.1136/bmjopen-2019-031486)
Supplement: Supplementary data [file bmjopen-2019-031486supp003.pdf]

Supplementary Data 3: Mean ranking of recovery domains for dichotomised baseline characteristics. Based on average rankings, the top ranked domain is highlighted. Whilst pain, walking and arm/hand function remained the priorities, for respondents who were male or had undergone surgery, or had impaired upper, lower or bladder function, arm/hand function had the top ranking. For patients with impaired upper limb sensation, walking function was the priority.

	Pain (SD)		Arm/Hand (SD)		Walking Function (SD)		Sexual Function (SD)		Bladder/ Bowel (SD)		Trunk Function (SD)		Sensation (SD)	
Gender, Male	3.1	(2.2)	3.2	(1.6)	2.6	(1.6)	4.2	(1.8)	5.3	(1.7)	4.4	(1.6)	4.2	(2.3)
Gender, Female	2.5	(2.0)	2.9	(1.4)	3.0	(1.7)	4.1	(1.6)	5.9	(1.6)	4.6	(1.5)	4.4	(2.0)
Undergone Surgery	2.8	(2.1)	3.0	(1.4)	2.6	(1.6)	4.1	(1.6)	5.8	(1.6)	4.6	(1.6)	4.5	(2.0)
Not Undergone Surgery	2.6	(2.0)	2.9	(1.5)	3.2	(1.7)	4.2	(1.7)	5.6	(1.7)	4.4	(1.5)	4.2	(2.1)
mJOA Upper Limb Function <3	2.9	(2.0)	2.1	(1.3)	2.5	(1.3)	3.7	(1.6)	6.0	(1.7)	4.4	(1.3)	4.2	(2.1)
mJOA Upper Limb Function >3	2.6	(2.0)	3.1	(1.5)	3.0	(1.7)	4.2	(1.7)	5.7	(1.7)	4.5	(1.6)	4.3	(2.1)
mJOA Lower Limb Function <4	2.8	(2.0)	2.9	(1.5)	2.4	(1.5)	4.1	(1.6)	5.7	(1.7)	4.6	(1.4)	4.3	(2.1)
mJOA Lower Limb Function >4	2.6	(2.1)	3.0	(1.5)	3.2	(1.7)	4.2	(1.7)	5.7	(1.7)	4.5	(1.6)	4.3	(2.0)
mJOA Upper Limb Sensation <2	2.7	(2.1)	2.6	(1.3)	3.0	(1.5)	4.2	(1.5)	5.9	(1.6)	4.5	(1.5)	4.2	(2.1)
mJOA Upper Limb Sensation >2	2.6	(2.0)	3.2	(1.6)	3.0	(1.8)	4.1	(1.8)	5.6	(1.7)	4.5	(1.6)	4.4	(2.1)
mJOA Bladder Function <2	2.6	(1.8)	2.8	(1.6)	2.5	(1.4)	3.6	(1.4)	6.0	(1.3)	4.7	(1.6)	4.7	(2.0)
mJOA Bladder Function >2	2.7	(2.1)	3.0	(1.5)	3.1	(1.7)	4.3	(1.7)	5.6	(1.7)	4.5	(1.5)	4.2	(2.1)
Length of Symptoms <3 years	2.9	(2.2)	3.0	(1.5)	3.1	(1.6)	4.2	(1.7)	5.6	(1.7)	4.6	(1.5)	4.1	(2.1)
Length of Symptoms >3 years	2.5	(1.9)	2.9	(1.4)	2.8	(1.7)	4.1	(1.7)	5.8	(1.6)	4.5	(1.5)	4.5	(2.1)
Best Limb Pain VAS <3	2.7	(2.1)	3.0	(1.5)	2.9	(1.7)	4.2	(1.7)	5.7	(1.7)	4.4	(1.5)	4.6	(2.0)
Best Limb Pain VAS >3	2.5	(2.0)	2.9	(1.5)	3.0	(1.6)	4.1	(1.6)	5.8	(1.6)	4.7	(1.6)	3.9	(2.2)
