# Supplementary material for: Recovery priorities in degenerative cervical myelopathy: a cross-sectional survey of an international, online community of patients
Source: BMJ Open. 2019 Oct 10;9(10):e031486. doi: 10.1136/bmjopen-2019-031486 (PMC6797315; doi:10.1136/bmjopen-2019-031486)
Supplement: Supplementary data [file bmjopen-2019-031486supp004.pdf]

Supplementary Data 4: Questionnaire. The questions relevant to this study were developed, piloted and embedded within an ongoing initiative investigating patient reporting of DCM. The questions pertaining to the data points required for this study are presented, including their question number and options for selection. Question 35 about age was the only question where respondents were asked to type in a specific answer. The answer format was electronically validated to require an integer, prompting users to specify to the nearest year.

Questions generating data points required for this study	
5	How long have you suffered with cervical myelopathy?<br>0-1 year<br>1-3 years<br>3-10 years<br>10-25 years<br>>25 years
7	Have you undergone surgery for cervical myelopathy?<br>Yes<br>No
23	Currently, please indicate the intensity of the current, best and worst pain affecting your arms or legs over the past 24h on a scale of 0 (no pain) to 10 (worst pain imaginable)<br>0<br>1<br>2<br>3<br>4<br>5<br>6<br>7<br>8<br>9<br>10
29	How does cervical myelopathy affect your arms and hands? *Choose the statement that best fits:*<br>- I am unable to move my hands<br>- I am unable to eat with a spoon but can move my hands<br>- I am unable to button my shirt but able to eat with a spoon<br>- I can button my shirt with great difficulty<br>- I can button my shirt with slight difficulty<br>- I do not have any trouble using my hands.

30	How does cervical myelopathy affect your legs? *Choose the statement that best fits:*<br><br>- I am completely unable to move my legs at all and have no feeling in my legs<br>- I have feeling in my legs but am not able to move them at all<br>- I can move my legs but am unable to walk<br>- I can walk on flat floor with a walking aid (cane or crutch)<br>- I can walk up and/or downstairs with the aid of a handrail<br>- I can walk up and/or downstairs without handrail but I notice moderate-to-significant lack of stability/feeling of imbalance when I walk<br>- I can walk unaided (no crutches, canes, walker) with smooth reciprocation (i.e. my legs move smoothly) but I still notice a mild lack of stability/feeling of imbalance when walking<br>- I can walk without any problems of imbalance or instability
31	How does cervical myelopathy affect your arms and hands? *Choose the statement that best fits: I have*<br><br>- complete loss of feeling in hands<br><br>- severe loss of feeling, or have pain in my hands<br><br>- mild loss of feeling in my hands<br><br>- no loss of feeling in my hands
32	How does cervical myelopathy affect your bladder? *Choose the statement that best fits:*<br><br>- I am completely unable to control urination<br><br>- I have marked difficulty controlling urination<br><br>- I have mild to moderate difficulty controlling urination<br><br>- I have no difficulty controlling urination

33	Effective medical research should target the needs of patients. The consequences of spinal cord injury can be classified into 7 different categories. In DCM, the patient priorities are not known.<br><br>For you as a patient, what are the research priorities for you (please rank where 1 is the most important and 7 is the least important)? What would you like researchers to focus on?<br><br>-Elimination of Pain<br>-Arm/Hand Function<br>-Walking Function<br>-Bladder/Bowel Function<br>-Sexual Function<br>-Upper Body/Trunk Function<br>-Normal Sensation
34	Are you male or female?<br><br>Male<br>Female
35	How old are you?
