# Supplementary material for: Recovery priorities in degenerative cervical myelopathy: a cross-sectional survey of an international, online community of patients
Source: BMJ Open. 2019 Oct 10;9(10):e031486. doi: 10.1136/bmjopen-2019-031486 (PMC6797315; doi:10.1136/bmjopen-2019-031486)
Supplement: Author's manuscript [file bmjopen-2019-031486.draft_revisions.pdf]

BMJ Open is committed to open peer review. As part of this commitment we make the peer review history of every article we publish publicly available.

When an article is published we post the peer reviewers' comments and the authors' responses online. We also post the versions of the paper that were used during peer review. These are the versions that the peer review comments apply to.

The versions of the paper that follow are the versions that were submitted during the peer review process. They are not the versions of record or the final published versions. They should not be cited or distributed as the published version of this manuscript.

BMJ Open is an open access journal and the full, final, typeset and author-corrected version of record of the manuscript is available on our site with no access controls, subscription charges or pay-per-view fees (http://bmjopen.bmj.com).

If you have any questions on BMJ Open's open peer review process please email info.bmjopen@bmj.com

BMJ Open

Recovery priorities of patients with degenerative cervical myelopathy

Journal:	*BMJ Open*
Manuscript ID	bmjopen-2019-031486
Article Type:	Research
Date Submitted by the Author:	26-May-2019
Complete List of Authors:	Davies, Benjamin; University of Cambridge, Department of Clinical Neurosurgery<br>Mowforth, Oliver; University of Cambridge, Division of Neurosurgery, Department of Clinical Neurosciences<br>Sadler, Iwan; Myelopathy.org<br>Aarabi, Bizhan; University of Maryland Baltimore, Division of Neurosurgery<br>Kwon, Brian; University of British Columbia<br>Kurpad, Shekar; Medical College of Wisconsin, Department of Neurosurgery<br>Harrop, James; Toronto Western Hospital,<br>Wilson, Jefferson R.; University of Toronto<br>Grossman, Robert; Houston Methodist Hospital<br>Fehlings, Michael G.; Toronto Western Hospital,<br>Kotter, Mark; University of Cambridge, Department of Clinical Neurosurgery; Ann McLaren Laboratory of Regenerative Medicine, Department of Clinical Neurosciences
Keywords:	Spine < ORTHOPAEDIC & TRAUMA SURGERY, NEUROSURGERY, Neurosurgery < SURGERY, Neurological pain < NEUROLOGY

SCHOLARONE™
Manuscripts

Recovery priorities of patients with degenerative cervical myelopathy

Benjamin M. Davies M.R.C.S B.Sc[1], Oliver D. Mowforth BA[1], Iwan Sadler[2], Bizhan Aarabi MD[3], Brian K. Kwon M.D. Ph.D F.R.C.S.C[4], Shekar N. Kurpad M.D Ph.D[5], James Harrop M.D.[6] Jefferson R. Wilson M.D. Ph.D[7], Robert Grossman M.D.[8], Michael G. Fehlings M.D. Ph.D F.R.C.S.C[9] , Mark R.N. Kotter M.D. M.Phil. Ph.D.[1,10]

1) Academic Neurosurgery Unit, Department of Clinical Neurosurgery, University of Cambridge, Cambridge, UK
2) DCM Sufferer and Director of Myelopathy Support, Myelopathy.org, Cambridge, UK
3) Division of Neurosurgery, Shock Trauma, University of Maryland, Baltimore, Maryland, USA
4) Division of Spine Surgery, Vancouver General Hospital, University of British Columbia, Vancouver, British Columbia, Canada
5) Department of Neurosurgery, Medical College of Wisconsin, Milwaukee, Wisconsin, USA
6) Division of Neurosurgery, Thomas Jefferson University Hospital, Philadelphia, Pennsylvania, USA
7) Division of Neurosurgery, Department of Surgery, University of Toronto, Toronto, Ontario, Canada
8) Division of Neurosurgery, Houston Methodist Hospital , Houston, Texas, USA
9) Division of Neurosurgery and Spine Program, Toronto Western Hospital, University Health Network, University of Toronto, Toronto, Ontario, Canada
10) Wellcome Trust & MRC Cambridge Stem Cell Institute, UK

Running title: Pain is the recovery priority in degenerative cervical myelopathy

Key words: cervical; myelopathy; spondylosis; spondylotic; stenosis; disc herniation; ossification posterior longitudinal ligament; degeneration; disability; recovery; questionnaire; priority setting; patient and public involvement; research wastage.

Main text word count*:* 2477

Reference count: 35

Funding: Research in the senior author's laboratory is supported by a core support grant from the Wellcome Trust and MRC to the Wellcome Trust-Medical Research Council Cambridge Stem Cell Institute. MRNK is supported by a NIHR Clinician Scientist Award. MGF is supported by the Halbert Chair in Neural Repair and Regeneration. Partial support for this work was obtained from the AOSpine Knowledge Forum in Spinal Cord Injury. The authors also acknowledge the AOSpine for their support of travel and meetings costs. The NIHR HTC, from the Brain Injury Medical Technology Cooperative, provided the funding for Google Adwords survey advertising.

Disclaimer: This report is independent research arising from a Clinician Scientist Award, CS-2015-15-023, supported by the National Institute for Health Research. The views expressed in this publication are those of the authors and not necessarily those of the NHS, the National Institute for Health Research or the Department of Health and Social Care.

Correspondence to:
Dr Mark Kotter
Anne McLaren Laboratory
Department of Clinical Neurosciences
WT MRC Cambridge Stem Cell Institute
West Forvie Building, Forvie Site
University of Cambridge
CB2 0SZ
Email: mrk25@cam.ac.uk
Telephone: +44 1223 763366
Fax: +44 1223 763350

For peer review only

Abstract

Objectives

To establish the recovery priorities of individuals suffering with degenerative cervical myelopathy (DCM).

Design

A cross-sectional, observational study.

Setting

Patients from across the world with a diagnosis of DCM accessed the survey over an 18-month period on Myelopathy.org, an international myelopathy charity.

Participants

481 individuals suffering from DCM completed the online survey fully.

Main outcome measures

Functional recovery domains were established through qualitative interviews and a consensus process. Individuals were asked about their disease characteristics, including limb pain (visual analogue scale) and functional disability (patient derived - modified Japanese Orthopaedic Association score). Individuals ranked recovery domains (arm and hand function, walking, upper body/trunk function, sexual function, elimination of pain, sensation and bladder/bowel function) in order of priority. Priorities were analysed as the modal first priority and mean ranking. The influence of demographics on selection was analysed, with significance $p<0.05$.

Results

Of 659 survey responses obtained, 481 were complete. Overall, pain was the most popular recovery priority (39.9%) of respondents, followed by walking (20.2%), sensation (11.9%) and arm and hand function (11.5%). Sexual function (5.7%), bladder and bowel (3.7%) or trunk function (3.5%) were chosen less frequently. When considering the average ranking of symptoms, whilst pain remained the priority (2.6±2.0), this was closely followed by walking (2.9±1.7) and arm/hand function (3.0 ±1.4). Sensation ranked much lower (4.3±2.1). With respect to disease characteristics, overall pain remained the recovery priority, with the exception of patients with greater walking impairment ($p<0.005$) who prioritised walking, even amongst patients with lower pain scores.

Conclusions

This is the first study investigating patient priorities in DCM. The patient priorities reported provide an important framework for future research and will help ensure that it is aligned with patient needs.

For peer review only

Strengths

- **Patient-Focussed Research.** The misalignment of researcher and patient objectives has been found to contribute to research wastage therefore recovery domains were agreed using a patient focus group, patients have been involved in all stages and are represented amongst the authors.
- **Large survey population, including both patients who have and have not undergone surgical treatment.** Not all DCM patients require surgery, but DCM research has largely reported on cohorts of less than 300 surgical DCM patients, therefore the broader sampling in this study is likely more representative.
- **Demographic data, including validated outcome tools, allowed subgroup analysis.** DCM can cause wide ranging disability, and a representation of this was captured using additional data points, in order to ascertain whether recovery priorities were generalisable.

Limitations

- **Self-selected population.** Patients who do not engage with Myelopathy.org would not have had the opportunity to complete the questionnaire.
- **Risk of inclusion of non-patients.** Respondents were asked to click through a description of DCM and confirm they had a diagnosis from a medical professional before completing the questionnaire.

For peer review only

Introduction

Degenerative cervical myelopathy [DCM] has been coined as an umbrella term for degenerative and congenital or acquired conditions of the cervical spine, such as spondylosis or ossification of the posterior longitudinal ligament (OPLL), which lead to symptomatic cord compression.[1] With an estimated prevalence of up to 5% in individuals above 40 years old,[2],[3] DCM is the most common cause of spinal cord dysfunction worldwide.[1] Given its degenerative aetiology and the rising age of the population, this incidence is expected to rise.[4]

The cervical spinal cord acts as a processor and conduit of information between the brain and the periphery. Its injury can therefore give rise to a range of possible symptoms.[1] These include pain, paraesthesia, weakness, unsteadiness, frequent falls, bladder or bowel dysfunction and impotence in men.[5] At early stages, individual symptoms may occur in isolation, but more typically occur in combination, especially as the disease advances.

At present, decompressive surgery is the only evidence-based treatment for DCM.[6]. Surgical decompression is able to halt the progression of symptoms and offer limited, albeit clinically relevant[7] improvements across a range of domains.[8],[9] However, due to the limited intrinsic capacity for the spinal cord to repair, most patients do not make a full recovery, and instead suffer lifelong disabilities.[9] As a consequence, unemployment and/or dependency is prevalent amongst individuals with DCM.[4],[10],[11] Moreover, a recent study has identified that DCM severely impacts quality of life with recorded SF-36, patient reported outcome scores amongst the lowest of all chronic disease.[12] Improving recovery is therefore a major unmet clinical need in DCM.[13]

Medical research is primarily designed by health care professionals. This bears the risk of not taking into account actual patient needs. The concept of 'research wastage' has emerged to depict healthcare research that does not yield actual or potential clinical benefit. In the 2014 *Lancet* series, Chalmers et al. estimated that as much as 85% of the US$240 billion expended on health research in 2010 was wasted and an important contributing factor was the misalignment of patient and clinician research objectives.[14],[15] As a consequence, several research funding bodies now advocate the involvement of patients in the design and conduct of research. This has demonstrable beneficial impact.[16] Patient and public involvement (PPI) plays a particularly important role in the National Institute for Health Research (NIHR).[17] In addition to participation and engagement in the research process, the involvement of patients in identifying relevant research topics and their prioritisation is particularly encouraged. Organisations such as the James Lind Alliance have

For peer review only

successfully brought together patients, professionals and industry in order to set research priorities, e.g. for spinal cord injury.[18] However, the research priorities for individuals suffering from DCM have not yet been assessed.

A recent systematic review of DCM research demonstrated a heavy focus on surgical technique.[19],[20] However, the research needs of patients with DCM and their priorities remain unknown. Moreover, as part of a core-outcomes initiative (REsearch Objectives and COmmon Date Elements in DCM [RECODE-DCM]) we have identified that outcome domains are not consistently reported in current clinical research.[19] The present project set out to establish the needs and priorities of individuals suffering from DCM. This will help to determine the outcome assessments that should be included in clinical research and to better direct future research.

For peer review only

Methods

Reporting adheres to the EQUATOR Network STROBE checklist[21].

Survey development

Patient and Public Involvement

Individuals with DCM and their caregivers were invited to attend the Myelopathy.org Patient and Public Involvement day, hosted at the University of Cambridge and captured by Cambridge TV in their documentary.[22] Myelopathy.org is an international, charitable organisation for individuals affected by or working with DCM. As part of the event, qualitative interviews (N=9) were used to establish relevant functional domains that affected quality of life of individuals with DCM. These were found to resemble domains previously reported by Anderson et al. (2004), who conducted a survey amongst patients with traumatic spinal cord injury asking them to rank seven domains of spinal cord function in order of priority for recovery.[23] Using this as a template but broadening ‘upper body/trunk strength and balance’ to upper body/trunk function, the following recovery domains were agreed by participants: elimination of pain, arm and hand function, walking, sexual function, upper body/trunk function, sensation and bladder/bowel function. For brevity, in this article they are referred to as arm/hand, walking, sexual function, pain, sensation, trunk and bladder/bowel.

These questions were embedded into an existing electronic survey initiative, developed using Survey Monkey (California, USA) and following the Checklist for Reporting Results of Internet E-Surveys (CHERRIES),[24] investigating patient reporting of DCM. This iteration was piloted by the lead investigators and a selection of individuals with DCM. Ethical approval was granted by the University of Cambridge. Study objectives were outlined on the initial page, including details of the host organization and estimated time required to complete the survey. This acted as the electronic consent, with continuation into the survey as agreement. Respondents were also presented with a description of DCM, including relevant synonyms, and required to confirm they suffered with the condition.

Respondents were asked to rank recovery domains in order of priority and provide details about their DCM. DCM characteristics included age, gender, history of surgery, best daily limb pain score (using a visual analogue scale), duration of symptoms and disease severity as measured using the self-reported, modified Japanese Orthopaedic Association [mJOA].[25] The mJOA is amongst the most commonly utilised assessments of disease severity[19],[20] and is fully validated.[26] It is a composite score based on upper limb function, lower limb function, upper limb sensation and bladder function. The score is valid for analysis in its entirety or per domain. All questions were mandatory, but respondents were not required to rank every recovery domain, on the basis that some domains may not

For peer review only - http://bmjopen.bmj.com/site/about/guidelines.xhtml

be a priority for them. The sequence of questions and order of responses was not altered from respondent to respondent.

Survey administration

The survey was accessed via a landing page on Myelopathy.org, allowing assessment of response rates using Google Analytics (California, USA). Individuals with DCM were recruited over an eighteen-month period. The recruitment process has been described in detail previously[27] but in short, the survey was advertised using Google Adwords (California, USA) and through Myelopathy.org and its social media outlets. The survey was voluntary and internet protocol addresses were used to prevent users submitting multiple responses. A missing data analysis was conducted between complete and incomplete survey responses to consider if particular subgroups were more likely to terminate early. Complete responders were defined as having provided answers for all aforementioned variables.

Analysis

Research priorities are presented using summary statistics, including average ranking and overall proportion of patients per domain. Domains which were not ranked by a respondent were omitted from these scores. For subgroup analysis, variables were dichotomised and thresholds were chosen based on the graphical distribution of responses and sample sizes. Categorical variables were compared using the Chi-Squared test. For continuous variables, the Shapiro-Wilks test was used to assess for parametric distribution of data sets. The Mann-Whitney U test was then used to compare the means of non-parametric distributions whilst a two-tailed T-test used to compare the means of parametric distributions. Pearson's correlations were performed to assess between group differences in characteristics, which could have influenced sub-group analysis. Significance was set at $p < 0.05$.

For peer review only

Results

Respondents

The survey was uniquely accessed 1463 times, with 659 visitors entering the survey (participation rate of 33%). A total of 481 responses contained complete data (completion rate 73%). A missing data analysis was conducted comparing incomplete and complete responses. Patients who completed the survey in full were more likely to have undergone surgery (p = 0.04), otherwise there was no statistical difference within variables of interest (Supplementary Data 2). Only complete responses were analysed in the present study. Of these responses domains were ranked more than 80% of the time: pain (400, 83%), sensation (428, 89%), walking (396, 82%), arm and hand (393, 82%), sexual (388, 81%), bladder and bowel (399, 83%) and trunk function (407, 85%).

On average respondents were more likely to be female (341, 70%) and suffer with moderate myelopathy (11.9 ±3.0) for between 3 and 10 years (181, 31%). Around half of patients (221, 46%) had undergone surgery. Overall respondent demographics are summarised in Table 1. Considering group differences, patients who had suffered from the disease for longer were more likely to have undergone surgery (p <0.01) and have worse myelopathy (-0.22, p <0.005). They were also more likely to suffer more pain (-0.14, p <0.01). Average pain scores were 3.1 (±2.4) for patients suffering with the disease for less than a year, rising to 4.5 (±3.0) for patients suffering for at least 10 years. There was no relationship between severity of myelopathy and pain scores (-0.04, p = 0.36). Between group differences are summarised in Supplementary Data 3.

Ranking of spinal cord dysfunction domains

Overall, pain was the most popular number one ranked recovery domain, chosen by 39.9% of respondents. This was followed by walking (20.2%), sensation (11.9%) and arm and hand function (11.5%). Sexual function (5.7%), bladder and bowel (3.7%) or trunk function (3.5%) were chosen less frequently. When considering the average ranking of symptoms, whilst pain remained the priority (2.6±2.0), this was closely followed by walking (2.9±1.7) and arm/hand function (3.0 ±1.4) (Figure 1). Sensation ranked lower (4.3±2.1).

Impact of baseline characteristics on ranking of spinal cord dysfunction domains

Respondents who had undergone surgery were more likely to prioritise walking (p < 0.005) and trunk function (p = 0.03), whereas patients who had not yet undergone surgery were more likely to prioritise upper limb function (p < 0.05) (Figure 2). Patients with poor upper limb or lower limb function were more likely to prioritise arm/hand recovery (p < 0.005) and

For peer review only

walking ($p < 0.005$) respectively (Figure 2). Overall, pain remained the priority, with the exception of patients with greater walking impairment ($p < 0.005$), even amongst patients with lower pain scores (Figure 2).

When considering the average rankings pain, arm/hand function and walking remained the top three recovery priorities (Figure 3). However, amongst the subcategories, the order of these priorities differed slightly (Supplementary Data 4). Patients who were male, or who had undergone surgery, or who had greater lower limb or bladder functional disability, prioritised recovery of walking, over pain and arm/hand function; patients with greater upper limb function or sensory disability prioritised recovery of arm/hand function over pain and walking.

When overall mJOA scores were considered to evaluate mild, moderate and severe patients,[6] no variation was seen in modal or average ranked priorities.

For peer review only

Discussion

This is the first study to systematically survey functional domains relevant to DCM and to ask patients to rank them in order of importance to their quality of life. The established priorities are likely to reflect symptom prevalence and their impact on day to day life.[23] The analysis of 481 completed answers demonstrated that pain, arm/hand function and walking emerge as the most important spinal cord dysfunction domains. Although based on averaged rankings, there were some subtle differences in ordering of these three domains. With the exception of patients with significant gait impairment, elimination of pain was the recovery priority independent of baseline characteristic.

These findings are surprising: functional disability (specifically recovery of arm/hand and walking function) has been and continues to be a focus for researchers, typically in response to surgery,[8] but more recently with a shift towards enhancing post-surgical recovery.[13],[27] In contrast, pain is not widely recognised as an important relevant domain. Our recent review of outcome reporting in DCM clinical trials demonstrated that the overwhelming majority of studies (90%) reported outcomes related to function, but only 27% of studies reported outcomes related to pain,[19] despite the fact that pain is a well-recognised feature of DCM,[5] which often improves following surgery.[11] The present findings highlight the fact that systematic research of patient needs is sorely lacking in DCM. A possible explanation for this discrepancy is that surgeons, who play a significant role in the management of DCM and predominate this research field, remain biased towards functional domains because pain is not a recognised indication for surgery in DCM.[6]

The priorities established in the present study differ from those of individuals suffering from spinal cord injury. Although pain is amongst the most prevalent symptoms of traumatic spinal cord injury,[28],[29] the "elimination of chronic pain" was considered to be a relatively low priority amongst those surveyed in Anderson's study[23] and a similar study by Kwon et al.[30] that focused on the priorities for SCI recovery after novel treatments (e.g. stem cells). Instead, quadriplegics prioritised arm/hand function, whilst paraplegics sexual and bladder/bowel function. These differences relate to their specific significance for patient independence and quality of life.

In DCM, the symptom burden is less well-described[31],[32] and the relationship between symptom burden or their significance with respect to quality of life in DCM has not been investigated. However, it would seem likely a similar relationship exists.

For peer review only

Limitations

Following recommendation by the James Lind Alliance, which was founded to support priority setting in research,[33],[34] the present survey was conducted online, as previously described.[27] Respondents belonged to a self-selecting group of individuals who were asked to confirm they had a diagnosis of DCM after being presented with an explanation of the disease for verification purposes. It is possible that some respondents did not have DCM. Reassuringly, respondent demographics were comparable to those of leading prospective surgical studies, with the exception of gender which was not shown to influence patient priorities[8],[9] (Supplementary Data 1). There are no such comparable series for non-surgical cohorts, but their inclusion provides a further valuable perspective.

The survey questions were not randomly sorted and therefore each respondent answered identical surveys with spinal cord function domains presented in the same order. The last domain assessed was sensation. In keeping with it being the most prevalent DCM symptom,[32] it featured most frequently in the responses, indicating that the order of domains was unlikely to have influenced the rankings. Moreover, answers to demographic questions, which followed the ranking of priorities on the survey, were required to define a complete response in order to be included in the present analysis. Priorities therefore were not influenced by incomplete answers.

Following the qualitative development work and the previous experience of Anderson et al., 2004, the pain domain was kept non-specific, asking patients to rank 'elimination of pain' as a recovery priority (Supplementary Data 2). In contrast however, the pain assessment focused on limb pain, which is classically felt to represent DCM-related pain.[5] Whilst this does not limit the implications of our findings as whole, their interpretation will require a better characterisation of pain in DCM in order to focus research appropriately as other pain foci are reported.[35]

Conclusion

The priorities reported in the present study identify functional domains that are relevant to the quality of life of DCM patients. They provide an important framework for future research and will serve as a valuable reference for the development of a core outcome set relevant to studies in DCM.

For peer review only

Table

Table 1: Summary of respondent demographics.

Respondent Demographics	
Age (Mean ± SD)	53.6 (9.8)
Male Gender (%)	140 (29)
Undergone Surgery (%)	221 (46)
Length of Symptoms (%)	
0 to 1 year	72 (15)
1 to 3 years	140 (29)
3 to 10 years	181 (38)
10 to 25 years	74 (15)
25+ years	14 (3)
mJOA (Mean +SD)	
Upper Limb Function	3.6 (1.0)
Walking	4.4 (1.5)
Upper Limb Sensation	1.7 (0.7)
Bladder Function	2.2 (1.0)
Total	11.9 (3.0)
VAS Limb Pain (Mean ± SD)	3.1 (2.6)

For peer review only

Figure Legends

Figure 1: Overall recovery priorities. The bar chart represents the first choice of patients and the line graph the average ranking for each domain (where the top ranking is 1). Pain was the overall first choice priority of patients, although when priority rankings were averaged, this was closely followed by walking and arm/hand function.

Figure 2: Impact of baseline characteristics on first choice recovery priority. The bar chart represents the first choice of patients. Significant between group differences are denoted by the * symbol. For simplicity, groups were dichotomised as follows: Duration <3years, mJOA upper limb function <3, mJOA lower limb function <4 or feeling <2, mJOA bladder/bowel function <2 and VAS limb pain <3. Those who had undergone surgery were more likely to choose trunk function (p=0.03) or walking function (p<0.005), whereas those who had not yet undergone surgery were more likely to choose arm/hand function (p<0.05). Equally patients with more impairment of walking (p<0.005) or arm/hand function (p<0.005) were more likely to prioritise these domains. Pain remained the priority even in patients reporting less pain.

Figure 3: Impact of baseline characteristics on recovery priority average rankings. The scatter plot represents the mean ranking for each subgroup investigated. The black line represents the overall average. Despite some discrepancies between subgroups, pain, arm/hand and walking function were consistently the top three priorities for patients. Bladder/bowel function was not a recovery priority.

For peer review only

Declaration Statement:

JH reports being a Medical Advisor for Depuy Synthes and Ethicon, being an Educational Speaker at Globus Medical and research funding from AO Spine. MGF reports consulting for Fortuna Fix. MRK declares a grant from the National Institute for Health Research, travel support from AO Spine and is founder of Myelopathy.org, the first charity for patients with cervical myelopathy. The remaining authors have nothing to declare.

Author Contributions Statement:

All authors were involved in the interpretation, drafting and final approval of the manuscript. Additionally, authors BMD, IS and MRK were involved in the conception, design and acquisition of data for this study, whilst authors BMD and ODM conducted the data analysis.

For peer review only

References:

1 Davies BM, Mowforth OD, Smith EK, *et al.* Degenerative cervical myelopathy. *BMJ* 2018;**360**:k186. doi:10.1136/bmj.k186

2 Kovalova I, Kerkovsky M, Kadanka Z, *et al.* Prevalence and Imaging Characteristics of Nonmyelopathic and Myelopathic Spondylotic Cervical Cord Compression. *Spine* 2016;**41**:1908–16. doi:10.1097/BRS.0000000000001842

3 Kadanka Z, Adamova B, Kerkovsky M, *et al.* Predictors of symptomatic myelopathy in degenerative cervical spinal cord compression. *Brain Behav* 2017;**7**. doi:10.1002/brb3.797

4 Nouri A, Tetreault L, Singh A, *et al.* Degenerative Cervical Myelopathy: Epidemiology, Genetics, and Pathogenesis. *Spine* 2015;**40**:E675-693. doi:10.1097/BRS.0000000000000913

5 Tracy JA, Bartleson JD. Cervical spondylotic myelopathy. *The Neurologist* 2010;**16**:176–87. doi:10.1097/NRL.0b013e3181da3a29

6 Fehlings MG, Tetreault LA, Riew KD, *et al.* A Clinical Practice Guideline for the Management of Patients With Degenerative Cervical Myelopathy: Recommendations for Patients With Mild, Moderate, and Severe Disease and Nonmyelopathic Patients With Evidence of Cord Compression. *Glob Spine J* 2017;**7**:70S-83S. doi:10.1177/2192568217701914

7 Tetreault L, Wilson JR, Kotter MRN, *et al.* Predicting the minimum clinically important difference in patients undergoing surgery for the treatment of degenerative cervical myelopathy. *Neurosurg Focus* 2016;**40**:E14. doi:10.3171/2016.3.FOCUS1665

8 Fehlings MG, Wilson JR, Kopjar B, *et al.* Efficacy and safety of surgical decompression in patients with cervical spondylotic myelopathy: results of the AOSpine North America prospective multi-center study. *J Bone Joint Surg Am* 2013;**95**:1651–8. doi:10.2106/JBJS.L.00589

9 Fehlings MG, Ibrahim A, Tetreault L, *et al.* A global perspective on the outcomes of surgical decompression in patients with cervical spondylotic myelopathy: results from the prospective multicenter AOSpine international study on 479 patients. *Spine* 2015;**40**:1322–8. doi:10.1097/BRS.0000000000000988

10 Al-Tamimi YZ, Guilfoyle M, Seeley H, *et al.* Measurement of long-term outcome in patients with cervical spondylotic myelopathy treated surgically. *Eur Spine J Off Publ Eur Spine Soc Eur Spinal Deform Soc Eur Sect Cerv Spine Res Soc* 2013;**22**:2552–7. doi:10.1007/s00586-013-2965-4

11 Fehlings MG, Tetreault LA, Kurpad S, *et al.* Change in Functional Impairment, Disability, and Quality of Life Following Operative Treatment for Degenerative Cervical Myelopathy: A Systematic Review and Meta-Analysis. *Glob Spine J* 2017;**7**:53S-69S. doi:10.1177/2192568217710137

12 Oh T, Lafage R, Lafage V, *et al.* Comparing Quality of Life in Cervical Spondylotic Myelopathy with Other Chronic Debilitating Diseases Using the Short Form Survey 36-Health Survey. *World Neurosurg* 2017;**106**:699–706. doi:10.1016/j.wneu.2016.12.124

For peer review only

13 Fehlings MG, Wilson JR, Karadimas SK, *et al.* Clinical evaluation of a neuroprotective drug in patients with cervical spondylotic myelopathy undergoing surgical treatment: design and rationale for the CSM-Protect trial. *Spine* 2013;**38**:S68-75. doi:10.1097/BRS.0b013e3182a7e9b0

14 Chalmers I, Glasziou P. Avoidable waste in the production and reporting of research evidence. *Lancet Lond Engl* 2009;**374**:86–9. doi:10.1016/S0140-6736(09)60329-9

15 Chalmers I, Bracken MB, Djulbegovic B, *et al.* How to increase value and reduce waste when research priorities are set. *Lancet Lond Engl* 2014;**383**:156–65. doi:10.1016/S0140-6736(13)62229-1

16 Brett J, Staniszewska S, Mockford C, *et al.* Mapping the impact of patient and public involvement on health and social care research: a systematic review. *Health Expect Int J Public Particip Health Care Health Policy* 2014;**17**:637–50. doi:10.1111/j.1369-7625.2012.00795.x

17 National Institute for Health Research. Patient and Public Involvement. www.nihr.ac.uk. https://www.nihr.ac.uk/patients-and-public/ (accessed Feb 16, 2018).

18 van Middendorp JJ, Allison HC, Ahuja S, *et al.* Top ten research priorities for spinal cord injury: the methodology and results of a British priority setting partnership. *Spinal Cord* 2016;**54**:341–6. doi:10.1038/sc.2015.199

19 Davies BM, McHugh M, Elgheriani A, *et al.* Reported Outcome Measures in Degenerative Cervical Myelopathy: A Systematic Review. *PloS One* 2016;**11**:e0157263. doi:10.1371/journal.pone.0157263

20 Davies BM, McHugh M, Elgheriani A, *et al.* The reporting of study and population characteristics in degenerative cervical myelopathy: A systematic review. *PloS One* 2017;**12**:e0172564. doi:10.1371/journal.pone.0172564

21 Elm E von, Altman DG, Egger M, *et al.* Strengthening the reporting of observational studies in epidemiology (STROBE) statement: guidelines for reporting observational studies. *BMJ* 2007;**335**:806–8. doi:10.1136/bmj.39335.541782.AD

22 Roberts E. Elemental Ideas: Cervical Spondylotic Myelopathy. http://www.myelopathy.org/the-csm-documentary.html.

23 Anderson KD. Targeting recovery: priorities of the spinal cord-injured population. *J Neurotrauma* 2004;**21**:1371–83. doi:10.1089/neu.2004.21.1371

24 Eysenbach G. Improving the Quality of Web Surveys: The Checklist for Reporting Results of Internet E-Surveys (CHERRIES). *J Med Internet Res* 2004;**6**. doi:10.2196/jmir.6.3.e34

25 Rhee JM, Shi WJ, Cyriac M, *et al.* The P-mJOA: A Patient-derived, Self-reported Outcome Instrument for Evaluating Cervical Myelopathy: Comparison with the mJOA. *Clin Spine Surg* 2018;**31**:E115–20. doi:10.1097/BSD.0000000000000591

26 Kopjar B, Tetreault L, Kalsi-Ryan S, *et al.* Psychometric properties of the modified Japanese Orthopaedic Association scale in patients with cervical spondylotic myelopathy. *Spine* 2015;**40**:E23-28. doi:10.1097/BRS.0000000000000648

For peer review only

27 Davies B, Kotter M. Lessons From Recruitment to an Internet-Based Survey for Degenerative Cervical Myelopathy: Comparison of Free and Fee-Based Methods. *JMIR Res Protoc* 2018;**7**:e18. doi:10.2196/resprot.6567

28 Dijkers M, Bryce T, Zanca J. Prevalence of chronic pain after traumatic spinal cord injury: a systematic review. *J Rehabil Res Dev* 2009;**46**:13–29.

29 Siddall PJ, Loeser JD. Pain following spinal cord injury. *Spinal Cord* 2001;**39**:63–73.

30 Kwon BK, Ghag A, Dvorak MF, *et al.* Expectations of benefit and tolerance to risk of individuals with spinal cord injury regarding potential participation in clinical trials. *J Neurotrauma* 2012;**29**:2727–37. doi:10.1089/neu.2012.2550

31 Zhang RJ, Shen CL, Zhang JX, *et al.* Clinical features and surgical outcomes of cervical spondylotic myelopathy in patients of different ages: a retrospective study. *Spinal Cord* 2018;**56**:7–13. doi:10.1038/sc.2017.91

32 Nouri A, Tetreault L, Dalzell K, *et al.* The Relationship Between Preoperative Clinical Presentation and Quantitative Magnetic Resonance Imaging Features in Patients With Degenerative Cervical Myelopathy. *Neurosurgery* 2017;**80**:121–8. doi:10.1227/NEU.0000000000001420

33 Petit-Zeman S, Firkins L, Scadding JW. The James Lind Alliance: tackling research mismatches. *Lancet Lond Engl* 2010;**376**:667–9. doi:10.1016/S0140-6736(10)60712-X

34 Finer S, Robb P, Cowan K, *et al.* Top ten research priorities for type 2 diabetes: results from the Diabetes UK-James Lind Alliance Priority Setting Partnership. *Lancet Diabetes Endocrinol* 2017;**5**:935–6. doi:10.1016/S2213-8587(17)30324-8

35 Schrot RJ, Mathew JS, Li Y, *et al.* Headache relief after anterior cervical discectomy: post hoc analysis of a randomized investigational device exemption trial: clinical article. *J Neurosurg Spine* 2014;**21**:217–22. doi:10.3171/2014.4.SPINE13669

For peer review only

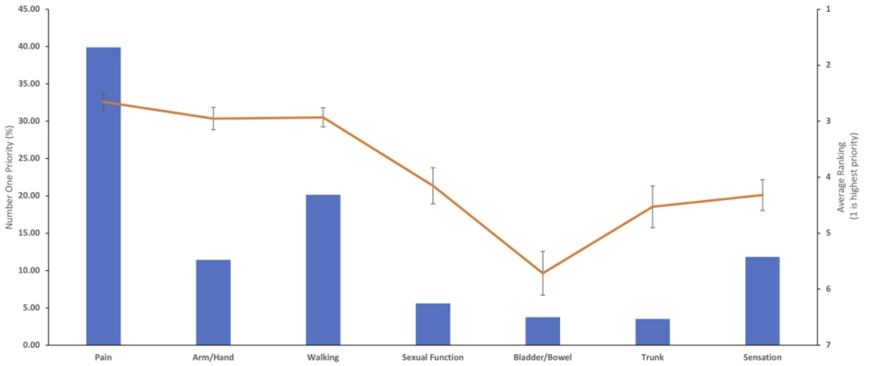

Figure 1: Overall recovery priorities. The bar chart represents the first choice of patients and the line graph the average ranking for each domain (where the top ranking is 1). Pain was the overall first choice priority of patients, although when priority rankings were averaged, this was closely followed by walking and arm/hand function.

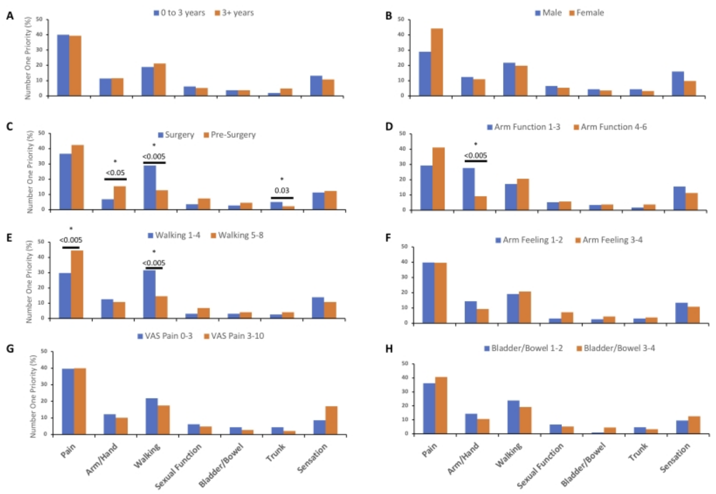

Figure 2: Impact of baseline characteristics on first choice recovery priority. The bar chart represents the first choice of patients. Significant between group differences are denoted by the * symbol. For simplicity, groups were dichotomised as follows: Duration <3years, mJOA upper limb function <3, mJOA lower limb function <4 or feeling <2, mJOA bladder/bowel function <2 and VAS limb pain <3. Those who had undergone surgery were more likely to choose trunk function (p=0.03) or walking function (p<0.005), whereas those who had not yet undergone surgery were more likely to choose arm/hand function (p<0.05). Equally patients with more impairment of walking (p<0.005) or arm/hand function (p<0.005) were more likely to prioritise these domains. Pain remained the priority even in patients reporting less pain.

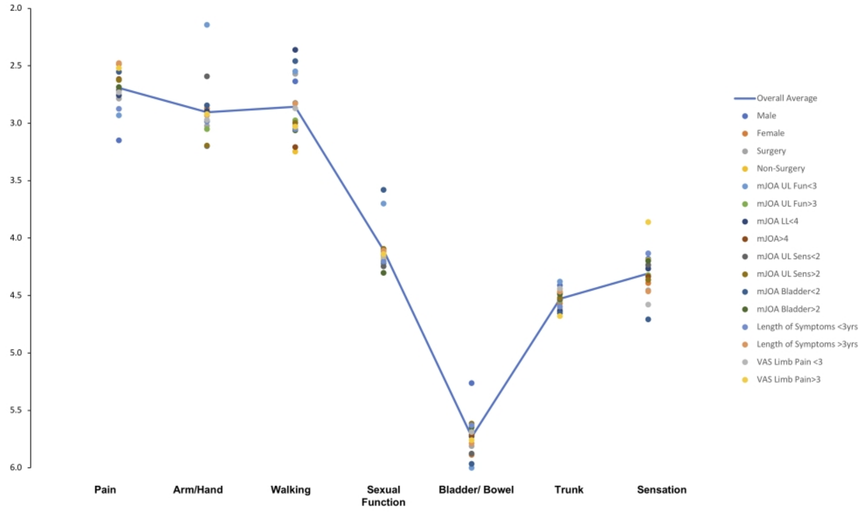

Figure 3: Impact of baseline characteristics on recovery priority average rankings. The scatter plot represents the mean ranking for each subgroup investigated. The black line represents the overall average. Despite some discrepancies between subgroups, pain, arm/hand and walking function were consistently the top three priorities for patients. Bladder/bowel function was not a recovery priority.

Supplementary Data 1: Results from the missing data analysis. Patients completing the survey in full, were more likely to have undergone surgery than those who did not ($p = 0.04$). Data is presented as mean +/- standard deviation, unless specified as a percentage. Numbers within brackets indicate data points for the respective variable with incomplete data.

	**INCOMPLETE SURVEYS (N<178)**	**COMPLETED SURVEYS (N=481)**	**P**
**%MALE**	16.3% (7/43)	28.9%	.076
**AGE**	55.1 +/-10.9 (43)	53.6 +/- 9.9	.344
**%SURGERY**	35.9% (46/128)	46.0%	.043
**LENGTH OF SYMPTOMS (YRS)**	19% (128)	73%	.304
**0-1**	18.0% (23)	15.0% (72)	
**1-3**	21.9% (28)	29.1% (140)	
**3-10**	35.2% (45)	37.6% (181)	
**10-25**	18.8% (24)	15.4% (74)	
**25+**	6.3% (8)	2.9% (14)	
**LIMB PAIN VAS**	3.6 +/- 1.9 (17)	3.7 +/- 2.5	.854
**MJOA**	12.2 +/- 3.2 (47)	11.9 +/- 3.0	.364

Supplementary Data 2: Questionnaire. The questions relevant to this study were developed, piloted and embedded within an ongoing initiative investigating patient reporting of DCM. The questions pertaining to the data points required for this study are presented, including their question number and options for selection. Question 35 about age was the only question where respondents were asked to type in a specific answer. The answer format was electronically validated to require an integer, prompting users to specify to the nearest year.

Questions generating data points required for this study	
5	How long have you suffered with cervical myelopathy?<br>0-1 year<br>1-3 years<br>3-10 years<br>10-25 years<br>>25 years
7	Have you undergone surgery for cervical myelopathy?<br>Yes<br>No
23	Currently, please indicate the intensity of the current, best and worst pain affecting your arms or legs over the past 24h on a scale of 0 (no pain) to 10 (worst pain imaginable)<br>0<br>1<br>2<br>3<br>4<br>5<br>6<br>7<br>8<br>9<br>10
29	How does cervical myelopathy affect your arms and hands? *Choose the statement that best fits: I am*<br>- Unable to move my hands<br>- Unable to eat with a spoon but am able to move my hands<br>- Unable to button my shirt but able to eat with a spoon<br>- Able to button my shirt with great difficulty<br>- Able to button my shirt with slight difficulty<br>- Not having any trouble using my hands.

For peer review only

30	How does cervical myelopathy affect your legs? *Choose the statement that best fits: I am*<br><br>- Completely unable to move legs at all and have no feeling in legs<br>- Having feeling in legs but not able to move them at all<br>- Able to move my legs but am unable to walk<br>- Able to walk on flat floor with a walking aid (cane or crutch)<br>- Able to walk up and/or downstairs with the aid of a handrail<br>- Able to walk up and/or downstairs without handrail but I notice moderate-to-significant lack of stability/feeling of imbalance when I walk<br>- Able to walk unaided (no crutches, canes, walker) with smooth reciprocation (ie, legs move smoothly) but I still notice mild lack of stability/feeling of imbalance when walking<br>- Able to walk without any problems of imbalance or instability
31	How does cervical myelopathy affect your arms and hands? *Choose the statement that best fits: I have*<br><br>- Complete loss of feeling in hands<br><br>- Severe loss of feeling, or have pain in my hands<br><br>- Mild loss of feeling in hands<br><br>- No loss of feeling in hands
32	How does cervical myelopathy affect your bladder? *Choose the statement that best fits: I am*<br><br>- Am completely unable to control urination<br><br>- Have marked difficulty controlling urination<br><br>- Have mild to moderate difficulty controlling urination<br><br>- No difficulty controlling urination
33	Effective medical research should target the needs of patients. The consequences of spinal cord injury can be classified into 7 different

For peer review only

	categories. In DCM, the patient priorities are not known.<br><br>For you as a patient, what are the research priorities for you (please rank where 1 is the most important and 7 is the least important)? What would you like researchers to focus on?<br><br>-Elimination of Pain<br>-Arm/Hand Function<br>-Walking Function<br>-Bladder/Bowel Function<br>-Sexual Function<br>-Upper Body/Trunk Function<br>-Normal Sensation
34	Are you male or female?<br><br>Male<br>Female
35	How old are you?

For peer review only

Supplementary Data 3: Summary of group differences between investigated variables. Highlighted cells represent significant differences in the proportion of respondents per group. Further analysis revealed that differences followed a logical course: patients who had had symptoms for longer or undergone surgery were more likely to have severe disease. In addition, patients with more severe disease were likely to have higher limb pain scores.

	N	Age (±SD)		Male (%)		Undergone Surgery (%)		Length of Symptoms <3yrs (%)		mJOA Upper Limb Function <3		mJOA Lower Limb Function <4		mJOA Upper Limb Sensation <2		mJOA Bladder Function <2		Mean VAS Limb Pain <3	
Gender, Male	140	55.8	10.3			64	46%	71	51%	20	14%	51	36%	50	36%	30	21%	88	63%
Gemder, Female	341	52.8	9.5			157	46%	141	41%	38	11%	107	31%	143	42%	75	22%	205	60%
Undergone Surgery	221	53.1	8.7	64	29%			83	38%	31	14%	90	41%	101	46%	53	24%	135	61%
Not Undergone Surgery	260	54	10.7	74	28%			129	50%	27	10%	68	26%	92	35%	53	20%	158	61%
mJOA Upper Limb Function <3	58	56.7	9.3	19	33%	31	53%	13	22%			46	79%	52	90%	29	50%	21	36%
mJOA Upper Limb Function >3	423	53.2	9.8	119	28%	190	45%	199	47%			112	26%	141	33%	76	18%	272	64%
mJOA Lower Limb Function <4	158	55.4	10.1	50	32%	90	57%	51	32%	46	29%			91	58%	61	39%	85	54%
mJOA Lower Limb Function >4	323	52.7	9.5	88	27%	131	41%	161	50%	12	4%			102	32%	44	14%	208	64%
mJOA Upper Limb Sensation <2	193	53.7	9.1	48	25%	101	52%	78	40%	52	27%	91	47%			67	35%	86	45%
mJOA Upper Limb Sensation >2	288	53.3	10.3	90	31%	120	42%	134	47%	6	2%	67	23%			38	13%	207	72%
mJOA Bladder Function <2	105	54	9.6	29	28%	53	50%	40	38%	29	28%	61	58%	67	64%			58	55%
mJOA Bladder Function >2	376	53.5	9.9	109	29%	168	45%	172	46%	29	8%	97	26%	126	34%			235	63%
Length of Symptoms <3 years	212	52.8	10.8	71	33%	83	39%			13	6%	51	24%	78	37%	40	19%	136	64%
Length of Symptoms >3 years	269	54.2	8.9	67	25%	138	51%			45	17%	107	40%	115	43%	65	24%	157	58%
Best Limb Pain VAS <3	293	53.3	11	88	30%	135	46%	136	46%	21	7%	85	29%	86	29%	58	20%		
Best Limb Pain VAS >3	188	53.6	8.6	52	28%	86	46%	76	40%	37	20%	73	39%	107	57%	47	25%		

Supplementary Data 4: Mean ranking for recovery domains for baseline characteristics. Based on average rankings, the top ranked domain is highlighted. Whilst pain, walking and arm/hand function remained the priorities, for respondents who were male or had undergone surgery, or had impaired upper, lower or bladder function, arm/hand function had the top ranking. For patients with impaired upper limb sensation, walking function was the priority.

	Pain (SD)		Walking (SD)		Arm/Hand Function (SD)		Sexual Function (SD)		Bladder/ Bowel (SD)		Trunk Function (SD)		Sensation (SD)	
Gender, Male	3.1	(2.2)	3.2	(1.6)	2.6	(1.6)	4.2	(1.8)	5.3	(1.7)	4.4	(1.6)	4.2	(2.3)
Gender, Female	2.5	(2.0)	2.9	(1.4)	3.0	(1.7)	4.1	(1.6)	5.9	(1.6)	4.6	(1.5)	4.4	(2.0)
Undergone Surgery	2.8	(2.1)	3.0	(1.4)	2.6	(1.6)	4.1	(1.6)	5.8	(1.6)	4.6	(1.6)	4.5	(2.0)
Not Undergone Surgery	2.6	(2.0)	2.9	(1.5)	3.2	(1.7)	4.2	(1.7)	5.6	(1.7)	4.4	(1.5)	4.2	(2.1)
mJOA Upper Limb Function <3	2.9	(2.0)	2.1	(1.3)	2.5	(1.3)	3.7	(1.6)	6.0	(1.7)	4.4	(1.3)	4.2	(2.1)
mJOA Upper Limb Function >3	2.6	(2.0)	3.1	(1.5)	3.0	(1.7)	4.2	(1.7)	5.7	(1.7)	4.5	(1.6)	4.3	(2.1)
mJOA Lower Limb Function <4	2.8	(2.0)	2.9	(1.5)	2.4	(1.5)	4.1	(1.6)	5.7	(1.7)	4.6	(1.4)	4.3	(2.1)
mJOA Lower Limb Function >4	2.6	(2.1)	3.0	(1.5)	3.2	(1.7)	4.2	(1.7)	5.7	(1.7)	4.5	(1.6)	4.3	(2.0)
mJOA Upper Limb Sensation <2	2.7	(2.1)	2.6	(1.3)		(1.5)	4.2	(1.5)	5.9	(1.6)	4.5	(1.5)	4.2	(2.1)
mJOA Upper Limb Sensation >2	2.6	(2.0)	3.2	(1.6)	3.0	(1.8)	4.1	(1.8)	5.6	(1.7)	4.5	(1.6)	4.4	(2.1)
mJOA Bladder Function <2	2.6	(1.8)	2.8	(1.6)	2.5	(1.4)	3.6	(1.4)	6.0	(1.3)	4.7	(1.6)	4.7	(2.0)
mJOA Bladder Function >2	2.7	(2.1)	3.0	(1.5)	3.1	(1.7)	4.3	(1.7)	5.6	(1.7)	4.5	(1.5)	4.2	(2.1)
Length of Symptoms <3 years	2.9	(2.2)	3.0	(1.5)	3.1	(1.6)	4.2	(1.7)	5.6	(1.7)	4.6	(1.5)	4.1	(2.1)
Length of Symptoms >3 years	2.5	(1.9)	2.9	(1.4)	2.8	(1.7)	4.1	(1.7)	5.8	(1.6)	4.5	(1.5)	4.5	(2.1)
Best Limb Pain VAS <3	2.7	(2.1)	3.0	(1.5)	2.9	(1.7)	4.2	(1.7)	5.7	(1.7)	4.4	(1.5)	4.6	(2.0)
Best Limb Pain VAS >3	2.5	(2.0)	2.9	(1.5)	3.0	(1.6)	4.1	(1.6)	5.8	(1.6)	4.7	(1.6)	3.9	(2.2)

For peer review only

STROBE Statement

Checklist of items that should be included in reports of observational studies

Section/Topic	Item No	Recommendation	Reported on Page No
**Title and abstract**	1	(*a*) Indicate the study's design with a commonly used term in the title or the abstract	2
		(*b*) Provide in the abstract an informative and balanced summary of what was done and what was found	2
**Introduction**			
Background/rationale	2	Explain the scientific background and rationale for the investigation being reported	4-5
Objectives	3	State specific objectives, including any prespecified hypotheses	5
**Methods**			
Study design	4	Present key elements of study design early in the paper	6-7
Setting	5	Describe the setting, locations, and relevant dates, including periods of recruitment, exposure, follow-up, and data collection	6-7
Participants	6	(*a*) *Cohort study*—Give the eligibility criteria, and the sources and methods of selection of participants. Describe methods of follow-up<br>*Case-control study*—Give the eligibility criteria, and the sources and methods of case ascertainment and control selection. Give the rationale for the choice of cases and controls<br>*Cross-sectional study*—Give the eligibility criteria, and the sources and methods of selection of participants	6-7
		(*b*) *Cohort study*—For matched studies, give matching criteria and number of exposed and unexposed<br>*Case-control study*—For matched studies, give matching criteria and the number of controls per case	
Variables	7	Clearly define all outcomes, exposures, predictors, potential confounders, and effect modifiers. Give diagnostic criteria, if applicable	6-7
Data sources/measurement	8*	For each variable of interest, give sources of data and details of methods of assessment (measurement). Describe comparability of assessment methods if there is more than one group	6-7
Bias	9	Describe any efforts to address potential sources of bias	6-7
Study size	10	Explain how the study size was arrived at	6-7
Quantitative variables	11	Explain how quantitative variables were handled in the analyses. If applicable, describe which groupings were chosen and why	6-7
Statistical methods	12	(*a*) Describe all statistical methods, including those used to control for confounding	7
		(*b*) Describe any methods used to examine subgroups and interactions	7
		(*c*) Explain how missing data were addressed	7
		(*d*) *Cohort study*—If applicable, explain how loss to follow-up was addressed<br>*Case-control study*—If applicable, explain how matching of cases and controls was addressed<br>*Cross-sectional study*—If applicable, describe analytical methods taking account of sampling strategy	7
		(*e*) Describe any sensitivity analyses	

For peer review only - http://bmjopen.bmj.com/site/about/guidelines.xhtml

Section/Topic	Item No	Recommendation	Reported on Page No
**Results**			
Participants	13*	(a) Report numbers of individuals at each stage of study—eg numbers potentially eligible, examined for eligibility, confirmed eligible, included in the study, completing follow-up, and analysed	8
		(b) Give reasons for non-participation at each stage	N/A
		(c) Consider use of a flow diagram	N/A
Descriptive data	14*	(a) Give characteristics of study participants (eg demographic, clinical, social) and information on exposures and potential confounders	8
		(b) Indicate number of participants with missing data for each variable of interest	Supplementary Data 2
		(c) *Cohort study*—Summarise follow-up time (eg, average and total amount)	N/A
Outcome data	15*	*Cohort study*—Report numbers of outcome events or summary measures over time	N/A
		Case-control study—Report numbers in each exposure category, or summary measures of exposure	N/A
		Cross-sectional study—Report numbers of outcome events or summary measures	12-13
Main results	16	(*a*) Give unadjusted estimates and, if applicable, confounder-adjusted estimates and their precision (eg, 95% confidence interval). Make clear which confounders were adjusted for and why they were included	8
		(*b*) Report category boundaries when continuous variables were categorized	Supplementary Data 1,3 & 4
		(*c*) If relevant, consider translating estimates of relative risk into absolute risk for a meaningful time period	N/A
Other analyses	17	Report other analyses done—eg analyses of subgroups and interactions, and sensitivity analyses	8-9
**Discussion**			
Key results	18	Summarise key results with reference to study objectives	10
Limitations	19	Discuss limitations of the study, taking into account sources of potential bias or imprecision. Discuss both direction and magnitude of any potential bias	11
Interpretation	20	Give a cautious overall interpretation of results considering objectives, limitations, multiplicity of analyses, results from similar studies, and other relevant evidence	10-11
Generalisability	21	Discuss the generalisability (external validity) of the study results	10-11
**Other Information**			
Funding	22	Give the source of funding and the role of the funders for the present study and, if applicable, for the original study on which the present article is based	1

**Give information separately for cases and controls in case-control studies and, if applicable, for exposed and unexposed groups in cohort and cross-sectional studies.*

For peer review only - http://bmjopen.bmj.com/site/about/guidelines.xhtml

Note: An Explanation and Elaboration article discusses each checklist item and gives methodological background and published examples of transparent reporting. The STROBE checklist is best used in conjunction with this article (freely available on the Web sites of PLoS Medicine at http://www.plosmedicine.org/, Annals of Internal Medicine at http://www.annals.org/, and Epidemiology at http://www.epidem.com/). Information on the STROBE Initiative is available at www.strobe-statement.org.

For peer review only

Recovery priorities in degenerative cervical myelopathy: a cross-sectional survey of an international, online community of patients

Journal:	*BMJ Open*
Manuscript ID	bmjopen-2019-031486.R1
Article Type:	Research
Date Submitted by the Author:	17-Jul-2019
Complete List of Authors:	Davies, Benjamin; University of Cambridge, Department of Clinical Neurosurgery<br>Mowforth, Oliver; University of Cambridge, Division of Neurosurgery, Department of Clinical Neurosciences<br>Sadler, Iwan; Myelopathy.org<br>Aarabi, Bizhan; University of Maryland Baltimore, Division of Neurosurgery<br>Kwon, Brian; University of British Columbia<br>Kurpad, Shekar; Medical College of Wisconsin, Department of Neurosurgery<br>Harrop, James; Toronto Western Hospital,<br>Wilson, Jefferson R.; University of Toronto<br>Grossman, Robert; Houston Methodist Hospital<br>Fehlings, Michael G.; Toronto Western Hospital,<br>Kotter, Mark; University of Cambridge, Department of Clinical Neurosurgery; Ann McLaren Laboratory of Regenerative Medicine, Department of Clinical Neurosciences
**Primary Subject Heading**:	Patient-centred medicine
Secondary Subject Heading:	Neurology
Keywords:	Spine < ORTHOPAEDIC & TRAUMA SURGERY, NEUROSURGERY, Neurosurgery < SURGERY, Neurological pain < NEUROLOGY

Recovery priorities in degenerative cervical myelopathy: a cross-sectional survey of an international, online community of patients

Benjamin M. Davies M.R.C.S B.Sc[1], Oliver D. Mowforth BA. MB. BChir.[1], Iwan Sadler[2], Bizhan Aarabi MD[3], Brian K. Kwon M.D. Ph.D F.R.C.S.C[4], Shekar N. Kurpad M.D Ph.D[5], James Harrop M.D.[6] Jefferson R. Wilson M.D. Ph.D[7], Robert Grossman M.D.[8], Michael G. Fehlings M.D. Ph.D F.R.C.S.C[9] , Mark R.N. Kotter M.D. M.Phil. Ph.D.[1,10]

1) Academic Neurosurgery Unit, Department of Clinical Neurosurgery, University of Cambridge, Cambridge, UK
2) DCM Sufferer and Director of Myelopathy Support, Myelopathy.org, Cambridge, UK
3) Division of Neurosurgery, Shock Trauma, University of Maryland, Baltimore, Maryland, USA
4) Division of Spine Surgery, Vancouver General Hospital, University of British Columbia, Vancouver, British Columbia, Canada
5) Department of Neurosurgery, Medical College of Wisconsin, Milwaukee, Wisconsin, USA
6) Division of Neurosurgery, Thomas Jefferson University Hospital, Philadelphia, Pennsylvania, USA
7) Division of Neurosurgery, Department of Surgery, University of Toronto, Toronto, Ontario, Canada
8) Division of Neurosurgery, Houston Methodist Hospital , Houston, Texas, USA
9) Division of Neurosurgery and Spine Program, Toronto Western Hospital, University Health Network, University of Toronto, Toronto, Ontario, Canada
10) Wellcome Trust & MRC Cambridge Stem Cell Institute, UK

Running title: Pain is the recovery priority in degenerative cervical myelopathy

Key words: cervical; myelopathy; spondylosis; spondylotic; stenosis; disc herniation; ossification posterior longitudinal ligament; degeneration; disability; recovery; questionnaire; priority setting; patient and public involvement; research wastage.

Main text word count*:* 2477

Reference count: 35

Funding: Research in the senior author's laboratory is supported by a core support grant from the Wellcome Trust and MRC to the Wellcome Trust-Medical Research Council Cambridge Stem Cell Institute. MRNK is supported by a NIHR Clinician Scientist Award. MGF is supported by the Halbert Chair in Neural Repair and Regeneration. Partial support for this work was obtained from the AOSpine Knowledge Forum in Spinal Cord Injury.
The authors also acknowledge the AOSpine for their support of travel and meetings costs.
The NIHR HTC, from the Brain Injury Medical Technology Cooperative, provided the funding for Google Adwords survey advertising.

Disclaimer: This report is independent research arising from a Clinician Scientist Award, CS-2015-15-023, supported by the National Institute for Health Research. The views expressed in this publication are those of the authors and not necessarily those of the NHS, the National Institute for Health Research or the Department of Health and Social Care.

Correspondence to:
Dr Mark Kotter
Anne McLaren Laboratory
Department of Clinical Neurosciences
WT MRC Cambridge Stem Cell Institute
West Forvie Building, Forvie Site
University of Cambridge
CB2 0SZ
Email: mrk25@cam.ac.uk
Telephone: +44 1223 763366
Fax: +44 1223 763350

For peer review only

Abstract

Objectives

To establish the recovery priorities of individuals suffering with degenerative cervical myelopathy (DCM).

Design

A cross-sectional, observational study.

Setting

Patients from across the world with a diagnosis of DCM accessed the survey over an 18-month period on Myelopathy.org, an international myelopathy charity.

Participants

481 individuals suffering from DCM completed the online survey fully.

Main outcome measures

Functional recovery domains were established through qualitative interviews and a consensus process. Individuals were asked about their disease characteristics, including limb pain (visual analogue scale) and functional disability (patient derived - modified Japanese Orthopaedic Association score). Individuals ranked recovery domains (arm and hand function, walking, upper body/trunk function, sexual function, elimination of pain, sensation and bladder/bowel function) in order of priority. Priorities were analysed as the modal first priority and mean ranking. The influence of demographics on selection was analysed, with significance $p<0.05$.

Results

Of 659 survey responses obtained, 481 were complete. Overall, pain was the most popular recovery priority (39.9%) of respondents, followed by walking (20.2%), sensation (11.9%) and arm and hand function (11.5%). Sexual function (5.7%), bladder and bowel (3.7%) or trunk function (3.5%) were chosen less frequently. When considering the average ranking of symptoms, whilst pain remained the priority (2.6±2.0), this was closely followed by walking (2.9±1.7) and arm/hand function (3.0 ±1.4). Sensation ranked much lower (4.3±2.1). With respect to disease characteristics, overall pain remained the recovery priority, with the exception of patients with greater walking impairment ($p<0.005$) who prioritised walking, even amongst patients with lower pain scores.

Conclusions

This is the first study investigating patient priorities in DCM. The patient priorities reported provide an important framework for future research and will help ensure that it is aligned with patient needs.

For peer review only

Strengths

- **This is the largest study of patient perspective in DCM to date and the first to consider patient recovery priorities**
- **This study is unique in reporting on both surgical and non-surgical DCM patients.**
- **This study includes a broad demographic representation of patients from across the globe and includes subgroup analysis.**

Limitations

- **This is an open-access, internet-based survey, a methodology which can lead to a sampling bias.**
- **Efforts to mitigate against sampling bias, alongside reassuring sub-group analysis suggest this risk is low.**

For peer review only

Introduction

Degenerative cervical myelopathy [DCM] has been coined as an umbrella term for degenerative and congenital or acquired conditions of the cervical spine, such as spondylosis or ossification of the posterior longitudinal ligament (OPLL), which lead to symptomatic cord compression.[1] With an estimated prevalence of up to 5% in individuals above 40 years old,[2],[3] DCM is the most common cause of spinal cord dysfunction worldwide.[1] Given its degenerative aetiology and the rising age of the population, this incidence is expected to rise.[4]

The cervical spinal cord acts as a processor and conduit of information between the brain and the periphery. Its injury can therefore give rise to a range of possible symptoms.[1] These include pain, paraesthesia, weakness, unsteadiness, frequent falls, bladder or bowel dysfunction and impotence in men.[5] At early stages, individual symptoms may occur in isolation, but more typically occur in combination, especially as the disease advances.

At present, decompressive surgery is the only evidence-based treatment for DCM.[6]. Surgical decompression is able to halt the progression of symptoms and offer limited, albeit clinically relevant[7] improvements across a range of domains.[8],[9] However, due to the limited intrinsic capacity for the spinal cord to repair, most patients do not make a full recovery, and instead suffer lifelong disabilities.[9] As a consequence, unemployment and/or dependency is prevalent amongst individuals with DCM.[4],[10],[11] Moreover, a recent study has identified that DCM severely impacts quality of life with recorded SF-36, patient reported outcome scores amongst the lowest of all chronic disease.[12] Improving recovery is therefore a major unmet clinical need in DCM.[13]

Medical research is primarily designed by health care professionals. This bears the risk of not taking into account actual patient needs. The concept of ‘research wastage’ has emerged to depict healthcare research that does not yield actual or potential clinical benefit. In the 2014 *Lancet* series, Chalmers et al. estimated that as much as 85% of the US$240 billion expended on health research in 2010 was wasted and an important contributing factor was the misalignment of patient and clinician research objectives.[14],[15] As a consequence, several research funding bodies now advocate the involvement of patients in the design and conduct of research. This has demonstrable beneficial impact.[16] Patient and public involvement (PPI) plays a particularly important role in the National Institute for Health Research (NIHR).[17] In addition to participation and engagement in the research process, the involvement of patients in identifying relevant research topics and their prioritisation is particularly encouraged. Organisations such as the James Lind Alliance have

For peer review only

For peer review only - http://bmjopen.bmj.com/site/about/guidelines.xhtml

successfully brought together patients, professionals and industry in order to set research priorities, e.g. for spinal cord injury.[18] However, the research priorities for individuals suffering from DCM have not yet been assessed.

A recent systematic review of DCM research demonstrated a heavy focus on surgical technique.[19],[20] However, the research needs of patients with DCM and their priorities remain unknown. Moreover, as part of a core-outcomes initiative (REsearch Objectives and COmmon Date Elements in DCM [RECODE-DCM]) we have identified that outcome domains are not consistently reported in current clinical research.[19]

In this study we sought to establish the recovery needs and priorities of individuals suffering from DCM. This will help to determine the outcome assessments that should be included in clinical research and to better direct future research.

For peer review only

Methods

Reporting adheres to the EQUATOR Network STROBE checklist[21].

Patient and Public Involvement

Individuals with DCM and their caregivers were invited to attend the Myelopathy.org Patient and Public Involvement day, hosted at the University of Cambridge and captured by Cambridge TV in their documentary.[22] Myelopathy.org is an international, charitable organisation for individuals affected by or working with DCM. As part of the event, qualitative interviews (N=9) were used to establish relevant functional domains that affected quality of life of individuals with DCM. These were found to resemble domains previously reported by Anderson et al. (2004), who conducted a survey amongst patients with traumatic spinal cord injury asking them to rank seven domains of spinal cord function in order of priority for recovery.[23] Using this as a template but broadening 'upper body/trunk strength and balance' to upper body/trunk function, the following recovery domains were agreed by participants: elimination of pain, arm and hand function, walking, sexual function, upper body/trunk function, sensation and bladder/bowel function. For brevity, in this article they are referred to as arm/hand, walking, sexual function, pain, sensation, trunk and bladder/bowel.

These questions were embedded into an existing electronic survey initiative, developed using Survey Monkey (California, USA) and following the Checklist for Reporting Results of Internet E-Surveys (CHERRIES),[24] investigating patient reporting of DCM. This iteration was piloted by the lead investigators and a selection of individuals with DCM. Ethical approval was granted by the University of Cambridge. Study objectives were outlined on the initial page, including details of the host organization and estimated time required to complete the survey. This acted as the electronic consent, with continuation into the survey as agreement. Respondents were also presented with a description of DCM, including relevant synonyms, and required to confirm they suffered with the condition.

Respondents were asked to rank recovery domains in order of priority and provide details about their DCM. DCM characteristics included age, gender, history of surgery, best daily limb pain score (using a visual analogue scale), duration of symptoms and disease severity as measured using the self-reported, patient-derived, modified Japanese Orthopaedic Association [**P**-mJOA]. [25] The modified Japanese Orthopaedic scale [mJOA] is amongst the most commonly utilised assessments of disease severity[19],[20] and is fully validated.[26] It is a composite score based on upper limb function, lower limb function, upper limb sensation and bladder function. The score is valid for analysis in its entirety or per domain. Originally developed as an investigator administered tool, it has recently been

For peer review only - http://bmjopen.bmj.com/site/about/guidelines.xhtml

adapted and validated for use by patients. [25] All questions were mandatory, but respondents were not required to rank every recovery domain, on the basis that some domains may not be a priority for them. The sequence of questions and order of responses was not altered from respondent to respondent.

Survey administration

The survey was accessed via a landing page on Myelopathy.org, allowing assessment of response rates using Google Analytics (California, USA). Individuals with DCM were recruited over an eighteen-month period. The recruitment process has been described in detail previously[27] but in short, the survey was advertised using Google Adwords (California, USA) and through Myelopathy.org and its social media outlets. The survey was voluntary and internet protocol addresses were used to prevent users submitting multiple responses. A missing data analysis was conducted between complete and incomplete survey responses to consider if particular subgroups were more likely to terminate early. Complete responders were defined as having provided answers for all aforementioned variables.

Analysis

Research priorities are presented using summary statistics, including average ranking and overall proportion of patients per domain. Domains which were not ranked by a respondent were omitted from these scores. For subgroup analysis, variables were dichotomised and thresholds were chosen based on the graphical distribution of responses and sample sizes. Categorical variables were compared using the Chi-Squared test. For continuous variables, the Shapiro-Wilks test was used to assess for parametric distribution of data sets. The Mann-Whitney U test was then used to compare the means of non-parametric distributions whilst a two-tailed T-test used to compare the means of parametric distributions. Pearson's correlations were performed to assess between group differences in characteristics, which could have influenced sub-group analysis. Significance was set at p <0.05.

For peer review only

Results

Respondents

The survey was uniquely accessed 1463 times, with 659 visitors entering the survey (participation rate of 33%). A total of 481 responses contained complete data (completion rate 73%). A missing data analysis was conducted comparing incomplete and complete responses. Patients who completed the survey in full were more likely to have undergone surgery (p = 0.04), otherwise there was no statistical difference within variables of interest (Supplementary Data 1). Only complete responses were analysed in the present study. Of these responses domains were ranked more than 80% of the time: pain (400, 83%), sensation (428, 89%), walking (396, 82%), arm and hand (393, 82%), sexual (388, 81%), bladder and bowel (399, 83%) and trunk function (407, 85%).

On average respondents were more likely to be female (341, 70%) and suffer with moderate myelopathy (11.9 ±3.0) for between 3 and 10 years (181, 31%). Around half of patients (221, 46%) had undergone surgery. Overall respondent demographics are summarised in Table 1. Considering group differences, patients who had suffered from the disease for longer were more likely to have undergone surgery (p <0.01) and have worse myelopathy (-0.22, p <0.005). They were also more likely to suffer more pain (-0.14, p <0.01). Average pain scores were 3.1 (±2.4) for patients suffering with the disease for less than a year, rising to 4.5 (±3.0) for patients suffering for at least 10 years. There was no relationship between severity of myelopathy and pain scores (-0.04, p = 0.36). Between group differences are summarised in Supplementary Data 2.

Ranking of spinal cord dysfunction domains

Overall, pain was the most popular number one ranked recovery domain, chosen by 39.9% of respondents. This was followed by walking (20.2%), sensation (11.9%) and arm and hand function (11.5%). Sexual function (5.7%), bladder and bowel (3.7%) or trunk function (3.5%) were chosen less frequently. When considering the average ranking of symptoms, whilst pain remained the priority (2.6±2.0), this was closely followed by walking (2.9±1.7) and arm/hand function (3.0 ±1.4) (Figure 1). Sensation ranked lower (4.3±2.1).

Impact of baseline characteristics on ranking of spinal cord dysfunction domains

Respondents who had undergone surgery were more likely to prioritise walking (p < 0.005) and trunk function (p = 0.03), whereas patients who had not yet undergone surgery were more likely to prioritise upper limb function (p < 0.05) (Figure 2). Patients with poor upper

For peer review only

limb or lower limb function were more likely to prioritise arm/hand recovery ($p < 0.005$) and walking ($p < 0.005$) respectively (Figure 2). Overall, pain remained the priority, with the exception of patients with greater walking impairment ($p < 0.005$), even amongst patients with lower pain scores (Figure 2).

When considering the average rankings pain, arm/hand function and walking remained the top three recovery priorities (Figure 3). However, amongst the subcategories, the order of these priorities differed slightly (Supplementary Data 3). Patients who were male, or who had undergone surgery, or who had greater lower limb or bladder functional disability, prioritised recovery of walking, over pain and arm/hand function; patients with greater upper limb function or sensory disability prioritised recovery of arm/hand function over pain and walking.

When overall P-mJOA scores were considered to evaluate mild, moderate and severe patients,[6] no variation was seen in modal or average ranked priorities.

For peer review only

Discussion

This is the first study to systematically survey functional domains relevant to DCM and to ask patients to rank them in order of importance to their quality of life. The established priorities are likely to reflect symptom prevalence and their impact on day to day life.[23] The analysis of 481 completed answers demonstrated that pain, arm/hand function and walking emerge as the most important spinal cord dysfunction domains. Although based on averaged rankings, there were some subtle differences in ordering of these three domains. With the exception of patients with significant gait impairment, elimination of pain was the recovery priority independent of baseline characteristic.

These findings are surprising: functional disability (specifically recovery of arm/hand and walking function) has been and continues to be a focus for researchers, typically in response to surgery,[8] but more recently with a shift towards enhancing post-surgical recovery.[13],[27] In contrast, pain is not widely recognised as an important relevant domain. Our recent review of outcome reporting in DCM clinical trials demonstrated that the overwhelming majority of studies (90%) reported outcomes related to function, but only 27% of studies reported outcomes related to pain,[19] despite the fact that pain is a well-recognised feature of DCM,[5] which often improves following surgery.[11] The present findings highlight the fact that systematic research of patient needs is sorely lacking in DCM. A possible explanation for this discrepancy is that surgeons, who play a significant role in the management of DCM and predominate this research field, remain biased towards functional domains because pain is not a recognised indication for surgery in DCM.[6]

The priorities established in the present study differ from those of individuals suffering from spinal cord injury. Although pain is amongst the most prevalent symptoms of traumatic spinal cord injury,[28],[29] the "elimination of chronic pain" was considered to be a relatively low priority amongst those surveyed in Anderson's study[23] and a similar study by Kwon et al.[30] that focused on the priorities for SCI recovery after novel treatments (e.g. stem cells). Instead, quadriplegics prioritised arm/hand function, whilst paraplegics sexual and bladder/bowel function. These differences relate to their specific significance for patient independence and quality of life.

In DCM, the symptom burden is less well-described[31],[32] and the relationship between symptom burden or their significance with respect to quality of life in DCM has not been investigated. However, it would seem likely a similar relationship exists.

For peer review only

Limitations

Following recommendation by the James Lind Alliance, which was founded to support priority setting in research,[33],[34] the present survey was conducted online, as previously described through a DCM charity, Myelopathy.org.[27] Respondents belonged to a self-selecting group of individuals who were asked to confirm they had a diagnosis of DCM by a medical professional, after being presented with an explanation of the disease for verification purposes. It is possible that some respondents did not have DCM. Reassuringly, respondent demographics were comparable to those of leading prospective surgical studies, with the exception of gender which was not shown to influence patient priorities[8],[9] (Supplementary Data 1). This likely reflects the recognised popularity of online health communities amongst females. There are no such comparable series for non-surgical cohorts, but their inclusion provides a further valuable perspective.

The survey questions were not randomly sorted and therefore each respondent answered identical surveys with spinal cord function domains presented in the same order. The last domain assessed was sensation. In keeping with it being the most prevalent DCM symptom,[32] it featured most frequently in the responses, indicating that the order of domains was unlikely to have influenced the rankings. Moreover, answers to demographic questions, which followed the ranking of priorities on the survey, were required to define a complete response in order to be included in the present analysis. Priorities therefore were not influenced by incomplete answers.

Following the qualitative development work and the previous experience of Anderson et al., 2004, the pain domain was kept non-specific, asking patients to rank ‘elimination of pain’ as a recovery priority (Supplementary Data 4). In contrast however, the pain assessment focused on limb pain, which is classically felt to represent DCM-related pain.[5] Whilst this does not limit the implications of our findings as whole, their interpretation will require a better characterisation of pain in DCM in order to focus research appropriately as other pain foci are reported.[35]

Conclusion

The priorities reported in the present study identify functional domains that are relevant to the quality of life of DCM patients. They provide an important framework for future research

For peer review only

and will serve as a valuable reference for the development of a core outcome set relevant to studies in DCM.

For peer review only

Table

Table 1: Summary of respondent demographics.

Respondent Demographics	
Age (Mean ± SD)	53.6 (9.8)
Male Gender (%)	140 (29)
Undergone Surgery (%)	221 (46)
Length of Symptoms (%)	
0 to 1 year	72 (15)
1 to 3 years	140 (29)
3 to 10 years	181 (38)
10 to 25 years	74 (15)
25+ years	14 (3)
P-mJOA (Mean +SD)	
Upper Limb Function	3.6 (1.0)
Walking	4.4 (1.5)
Upper Limb Sensation	1.7 (0.7)
Bladder Function	2.2 (1.0)
Total	11.9 (3.0)
VAS Limb Pain (Mean ± SD)	3.1 (2.6)

For peer review only

Figure Legends

Figure 1: Overall recovery priorities. The bar chart represents the first choice of patients and the line graph the average ranking for each domain (where the top ranking is 1). Pain was the overall first choice priority of patients, although when priority rankings were averaged, this was closely followed by walking and arm/hand function.

Figure 2: Impact of baseline characteristics on first choice recovery priority. The bar chart represents the first choice of patients. Significant between group differences are denoted by the * symbol. For simplicity, groups were dichotomised as follows: Duration <3years, P-mJOA upper limb function <3, P-mJOA lower limb function <4 or feeling <2, P-mJOA bladder/bowel function <2 and VAS limb pain <3. Those who had undergone surgery were more likely to choose trunk function (p=0.03) or walking function (p<0.005), whereas those who had not yet undergone surgery were more likely to choose arm/hand function (p<0.05). Equally patients with more impairment of walking (p<0.005) or arm/hand function (p<0.005) were more likely to prioritise these domains. Pain remained the priority even in patients reporting less pain.

Figure 3: Impact of baseline characteristics on recovery priority average rankings. The scatter plot represents the mean ranking for each subgroup investigated. The blue line represents the overall average. Despite some discrepancies between subgroups, pain, arm/hand and walking function were consistently the top three priorities for patients. Bladder/bowel function was not a recovery priority.

For peer review only

Declaration Statement:

JH reports being a Medical Advisor for Depuy Synthes and Ethicon, being an Educational Speaker at Globus Medical and research funding from AO Spine. MGF reports consulting for Fortuna Fix. MRK declares a grant from the National Institute for Health Research, travel support from AO Spine and is founder of Myelopathy.org, the first charity for patients with cervical myelopathy. The remaining authors have nothing to declare.

Author Contributions Statement:

BD, OM, IS, BA, BK, SK, JH, JW, RG, MF and MK were involved in the interpretation, drafting and final approval of the manuscript. Additionally, authors BD, IS and MK were involved in the conception, design and acquisition of data for this study, whilst authors BD and OM conducted the data analysis.

Data Availability Statement:

All relevant data to the study are included in the article or uploaded as supplementary information. No additional data are available.

For peer review only

For peer review only

References:

1 Davies BM, Mowforth OD, Smith EK, *et al.* Degenerative cervical myelopathy. *BMJ* 2018;**360**:k186. doi:10.1136/bmj.k186

2 Kovalova I, Kerkovsky M, Kadanka Z, *et al.* Prevalence and Imaging Characteristics of Nonmyelopathic and Myelopathic Spondylotic Cervical Cord Compression. *Spine* 2016;**41**:1908–16. doi:10.1097/BRS.0000000000001842

3 Kadanka Z, Adamova B, Kerkovsky M, *et al.* Predictors of symptomatic myelopathy in degenerative cervical spinal cord compression. *Brain Behav* 2017;**7**. doi:10.1002/brb3.797

4 Nouri A, Tetreault L, Singh A, *et al.* Degenerative Cervical Myelopathy: Epidemiology, Genetics, and Pathogenesis. *Spine* 2015;**40**:E675-693. doi:10.1097/BRS.0000000000000913

5 Tracy JA, Bartleson JD. Cervical spondylotic myelopathy. *The Neurologist* 2010;**16**:176–87. doi:10.1097/NRL.0b013e3181da3a29

6 Fehlings MG, Tetreault LA, Riew KD, *et al.* A Clinical Practice Guideline for the Management of Patients With Degenerative Cervical Myelopathy: Recommendations for Patients With Mild, Moderate, and Severe Disease and Nonmyelopathic Patients With Evidence of Cord Compression. *Glob Spine J* 2017;**7**:70S-83S. doi:10.1177/2192568217701914

7 Tetreault L, Wilson JR, Kotter MRN, *et al.* Predicting the minimum clinically important difference in patients undergoing surgery for the treatment of degenerative cervical myelopathy. *Neurosurg Focus* 2016;**40**:E14. doi:10.3171/2016.3.FOCUS1665

8 Fehlings MG, Wilson JR, Kopjar B, *et al.* Efficacy and safety of surgical decompression in patients with cervical spondylotic myelopathy: results of the AOSpine North America prospective multi-center study. *J Bone Joint Surg Am* 2013;**95**:1651–8. doi:10.2106/JBJS.L.00589

9 Fehlings MG, Ibrahim A, Tetreault L, *et al.* A global perspective on the outcomes of surgical decompression in patients with cervical spondylotic myelopathy: results from the prospective multicenter AOSpine international study on 479 patients. *Spine* 2015;**40**:1322–8. doi:10.1097/BRS.0000000000000988

10 Al-Tamimi YZ, Guilfoyle M, Seeley H, *et al.* Measurement of long-term outcome in patients with cervical spondylotic myelopathy treated surgically. *Eur Spine J Off Publ Eur Spine Soc Eur Spinal Deform Soc Eur Sect Cerv Spine Res Soc* 2013;**22**:2552–7. doi:10.1007/s00586-013-2965-4

11 Fehlings MG, Tetreault LA, Kurpad S, *et al.* Change in Functional Impairment, Disability, and Quality of Life Following Operative Treatment for Degenerative Cervical Myelopathy: A Systematic Review and Meta-Analysis. *Glob Spine J* 2017;**7**:53S-69S. doi:10.1177/2192568217710137

12 Oh T, Lafage R, Lafage V, *et al.* Comparing Quality of Life in Cervical Spondylotic Myelopathy with Other Chronic Debilitating Diseases Using the Short Form Survey 36-Health Survey. *World Neurosurg* 2017;**106**:699–706. doi:10.1016/j.wneu.2016.12.124

For peer review only

13 Fehlings MG, Wilson JR, Karadimas SK, *et al.* Clinical evaluation of a neuroprotective drug in patients with cervical spondylotic myelopathy undergoing surgical treatment: design and rationale for the CSM-Protect trial. *Spine* 2013;**38**:S68-75. doi:10.1097/BRS.0b013e3182a7e9b0

14 Chalmers I, Glasziou P. Avoidable waste in the production and reporting of research evidence. *Lancet Lond Engl* 2009;**374**:86–9. doi:10.1016/S0140-6736(09)60329-9

15 Chalmers I, Bracken MB, Djulbegovic B, *et al.* How to increase value and reduce waste when research priorities are set. *Lancet Lond Engl* 2014;**383**:156–65. doi:10.1016/S0140-6736(13)62229-1

16 Brett J, Staniszewska S, Mockford C, *et al.* Mapping the impact of patient and public involvement on health and social care research: a systematic review. *Health Expect Int J Public Particip Health Care Health Policy* 2014;**17**:637–50. doi:10.1111/j.1369-7625.2012.00795.x

17 National Institute for Health Research. Patient and Public Involvement. www.nihr.ac.uk. https://www.nihr.ac.uk/patients-and-public/ (accessed Feb 16, 2018).

18 van Middendorp JJ, Allison HC, Ahuja S, *et al.* Top ten research priorities for spinal cord injury: the methodology and results of a British priority setting partnership. *Spinal Cord* 2016;**54**:341–6. doi:10.1038/sc.2015.199

19 Davies BM, McHugh M, Elgheriani A, *et al.* Reported Outcome Measures in Degenerative Cervical Myelopathy: A Systematic Review. *PloS One* 2016;**11**:e0157263. doi:10.1371/journal.pone.0157263

20 Davies BM, McHugh M, Elgheriani A, *et al.* The reporting of study and population characteristics in degenerative cervical myelopathy: A systematic review. *PloS One* 2017;**12**:e0172564. doi:10.1371/journal.pone.0172564

21 Elm E von, Altman DG, Egger M, *et al.* Strengthening the reporting of observational studies in epidemiology (STROBE) statement: guidelines for reporting observational studies. *BMJ* 2007;**335**:806–8. doi:10.1136/bmj.39335.541782.AD

22 Roberts E. Elemental Ideas: Cervical Spondylotic Myelopathy. http://www.myelopathy.org/the-csm-documentary.html.

23 Anderson KD. Targeting recovery: priorities of the spinal cord-injured population. *J Neurotrauma* 2004;**21**:1371–83. doi:10.1089/neu.2004.21.1371

24 Eysenbach G. Improving the Quality of Web Surveys: The Checklist for Reporting Results of Internet E-Surveys (CHERRIES). *J Med Internet Res* 2004;**6**. doi:10.2196/jmir.6.3.e34

25 Rhee JM, Shi WJ, Cyriac M, *et al.* The P-mJOA: A Patient-derived, Self-reported Outcome Instrument for Evaluating Cervical Myelopathy: Comparison with the mJOA. *Clin Spine Surg* 2018;**31**:E115–20. doi:10.1097/BSD.0000000000000591

26 Kopjar B, Tetreault L, Kalsi-Ryan S, *et al.* Psychometric properties of the modified Japanese Orthopaedic Association scale in patients with cervical spondylotic myelopathy. *Spine* 2015;**40**:E23-28. doi:10.1097/BRS.0000000000000648

For peer review only

27 Davies B, Kotter M. Lessons From Recruitment to an Internet-Based Survey for Degenerative Cervical Myelopathy: Comparison of Free and Fee-Based Methods. *JMIR Res Protoc* 2018;**7**:e18. doi:10.2196/resprot.6567

28 Dijkers M, Bryce T, Zanca J. Prevalence of chronic pain after traumatic spinal cord injury: a systematic review. *J Rehabil Res Dev* 2009;**46**:13–29.

29 Siddall PJ, Loeser JD. Pain following spinal cord injury. *Spinal Cord* 2001;**39**:63–73.

30 Kwon BK, Ghag A, Dvorak MF, *et al.* Expectations of benefit and tolerance to risk of individuals with spinal cord injury regarding potential participation in clinical trials. *J Neurotrauma* 2012;**29**:2727–37. doi:10.1089/neu.2012.2550

31 Zhang RJ, Shen CL, Zhang JX, *et al.* Clinical features and surgical outcomes of cervical spondylotic myelopathy in patients of different ages: a retrospective study. *Spinal Cord* 2018;**56**:7–13. doi:10.1038/sc.2017.91

32 Nouri A, Tetreault L, Dalzell K, *et al.* The Relationship Between Preoperative Clinical Presentation and Quantitative Magnetic Resonance Imaging Features in Patients With Degenerative Cervical Myelopathy. *Neurosurgery* 2017;**80**:121–8. doi:10.1227/NEU.0000000000001420

33 Petit-Zeman S, Firkins L, Scadding JW. The James Lind Alliance: tackling research mismatches. *Lancet Lond Engl* 2010;**376**:667–9. doi:10.1016/S0140-6736(10)60712-X

34 Finer S, Robb P, Cowan K, *et al.* Top ten research priorities for type 2 diabetes: results from the Diabetes UK-James Lind Alliance Priority Setting Partnership. *Lancet Diabetes Endocrinol* 2017;**5**:935–6. doi:10.1016/S2213-8587(17)30324-8

35 Schrot RJ, Mathew JS, Li Y, *et al.* Headache relief after anterior cervical discectomy: post hoc analysis of a randomized investigational device exemption trial: clinical article. *J Neurosurg Spine* 2014;**21**:217–22. doi:10.3171/2014.4.SPINE13669

For peer review only

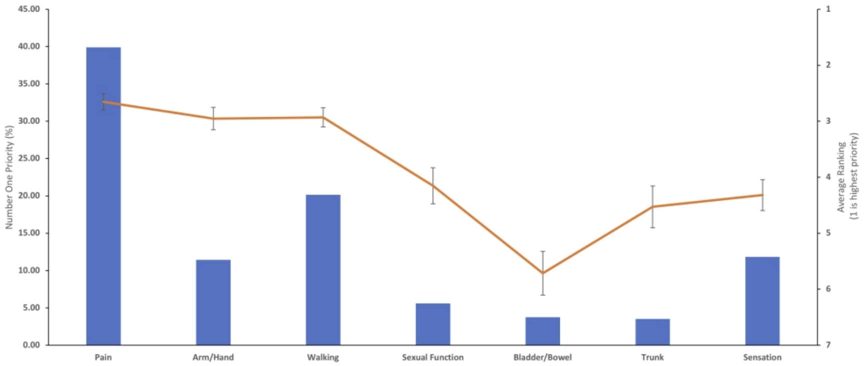

Figure 1: Overall recovery priorities. The bar chart represents the first choice of patients and the line graph the average ranking for each domain (where the top ranking is 1). Pain was the overall first choice priority of patients, although when priority rankings were averaged, this was closely followed by walking and arm/hand function.

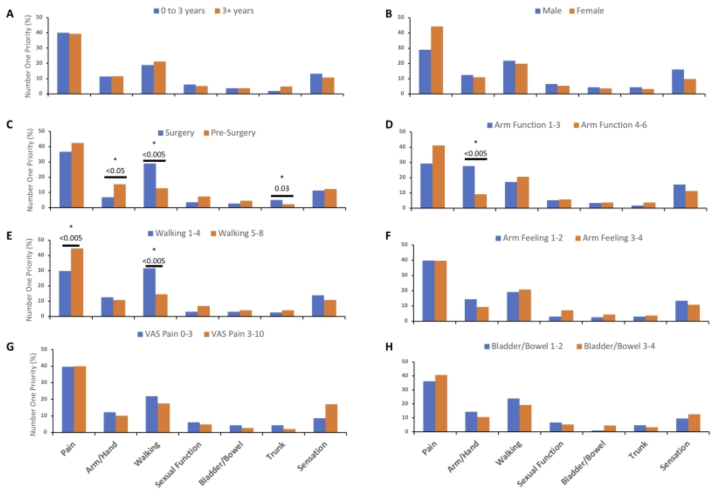

Figure 2: Impact of baseline characteristics on first choice recovery priority. The bar chart represents the first choice of patients. Significant between group differences are denoted by the * symbol. For simplicity, groups were dichotomised as follows: Duration <3years, mJOA upper limb function <3, mJOA lower limb function <4 or feeling <2, mJOA bladder/bowel function <2 and VAS limb pain <3. Those who had undergone surgery were more likely to choose trunk function (p=0.03) or walking function (p<0.005), whereas those who had not yet undergone surgery were more likely to choose arm/hand function (p<0.05). Equally patients with more impairment of walking (p<0.005) or arm/hand function (p<0.005) were more likely to prioritise these domains. Pain remained the priority even in patients reporting less pain.

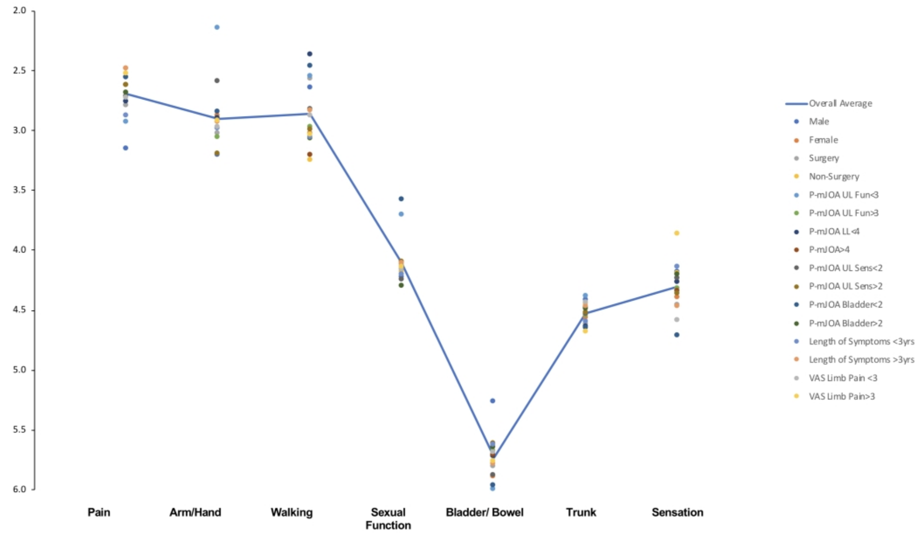

Figure 3: Impact of baseline characteristics on recovery priority average rankings. The scatter plot represents the mean ranking for each subgroup investigated. The black line represents the overall average. Despite some discrepancies between subgroups, pain, arm/hand and walking function were consistently the top three priorities for patients. Bladder/bowel function was not a recovery priority.

Supplementary Data 1: Results from the missing data analysis. Patients completing the survey in full, were more likely to have undergone surgery than those who did not (*p* = 0.04). Data is presented as mean +/- standard deviation, unless specified as a percentage. Numbers within brackets indicate data points for the respective variable with incomplete data.

	**INCOMPLETE SURVEYS (N<178)**	**COMPLETED SURVEYS (N=481)**	**P**
**%MALE**	16.3% (7/43)	28.9%	.076
**AGE**	55.1 +/-10.9 (43)	53.6 +/- 9.9	.344
**%SURGERY**	35.9% (46/128)	46.0%	.043
**LENGTH OF SYMPTOMS (YRS)**	19% (128)	73%	.304
**0-1**	18.0% (23)	15.0% (72)	
**1-3**	21.9% (28)	29.1% (140)	
**3-10**	35.2% (45)	37.6% (181)	
**10-25**	18.8% (24)	15.4% (74)	
**25+**	6.3% (8)	2.9% (14)	
**LIMB PAIN VAS**	3.6 +/- 1.9 (17)	3.7 +/- 2.5	.854
**MJOA**	12.2 +/- 3.2 (47)	11.9 +/- 3.0	.364

For peer review only - http://bmjopen.bmj.com/site/about/guidelines.xhtml

Supplementary Data 2: Summary of group differences between investigated variables. Highlighted cells represent significant differences in the proportion of respondents per group. Further analysis revealed that differences followed a logical course: patients who had had symptoms for longer or undergone surgery were more likely to have severe disease. In addition, patients with more severe disease were likely to have higher limb pain scores.

	N	Age (±SD)		Male (%)		Undergone Surgery (%)		Length of Symptoms <3yrs (%)		mJOA Upper Limb Function <3		mJOA Lower Limb Function <4		mJOA Upper Limb Sensation <2		mJOA Bladder Function <2		Mean VAS Limb Pain <3	
Gender, Male	140	55.8	10.3			64	46%	71	51%	20	14%	51	36%	50	36%	30	21%	88	63%
Gemder, Female	341	52.8	9.5			157	46%	141	41%	38	11%	107	31%	143	42%	75	22%	205	60%
Undergone Surgery	221	53.1	8.7	64	29%			83	38%	31	14%	90	41%	101	46%	53	24%	135	61%
Not Undergone Surgery	260	54	10.7	74	28%			129	50%	27	10%	68	26%	92	35%	53	20%	158	61%
mJOA Upper Limb Function <3	58	56.7	9.3	19	33%	31	53%	13	22%			46	79%	52	90%	29	50%	21	36%
mJOA Upper Limb Function >3	423	53.2	9.8	119	28%	190	45%	199	47%			112	26%	141	33%	76	18%	272	64%
mJOA Lower Limb Function <4	158	55.4	10.1	50	32%	90	57%	51	32%	46	29%			91	58%	61	39%	85	54%
mJOA Lower Limb Function >4	323	52.7	9.5	88	27%	131	41%	161	50%	12	4%			102	32%	44	14%	208	64%
mJOA Upper Limb Sensation <2	193	53.7	9.1	48	25%	101	52%	78	40%	52	27%	91	47%			67	35%	86	45%
mJOA Upper Limb Sensation >2	288	53.3	10.3	90	31%	120	42%	134	47%	6	2%	67	23%			38	13%	207	72%
mJOA Bladder Function <2	105	54	9.6	29	28%	53	50%	40	38%	29	28%	61	58%	67	64%			58	55%
mJOA Bladder Function >2	376	53.5	9.9	109	29%	168	45%	172	46%	29	8%	97	26%	126	34%			235	63%
Length of Symptoms <3 years	212	52.8	10.8	71	33%	83	39%			13	6%	51	24%	78	37%	40	19%	136	64%
Length of Symptoms >3 years	269	54.2	8.9	67	25%	138	51%			45	17%	107	40%	115	43%	65	24%	157	58%
Best Limb Pain VAS <3	293	53.3	11	88	30%	135	46%	136	46%	21	7%	85	29%	86	29%	58	20%		
Best Limb Pain VAS >3	188	53.6	8.6	52	28%	86	46%	76	40%	37	20%	73	39%	107	57%	47	25%		

Supplementary Data 3: Mean ranking for recovery domains for baseline characteristics. Based on average rankings, the top ranked domain is highlighted. Whilst pain, walking and arm/hand function remained the priorities, for respondents who were male or had undergone surgery, or had impaired upper, lower or bladder function, arm/hand function had the top ranking. For patients with impaired upper limb sensation, walking function was the priority.

	Pain (SD)		Walking (SD)		Arm/Hand Function (SD)		Sexual Function (SD)		Bladder/ Bowel (SD)		Trunk Function (SD)		Sensation (SD)	
Gender, Male	3.1	(2.2)	3.2	(1.6)	2.6	(1.6)	4.2	(1.8)	5.3	(1.7)	4.4	(1.6)	4.2	(2.3)
Gender, Female	2.5	(2.0)	2.9	(1.4)	3.0	(1.7)	4.1	(1.6)	5.9	(1.6)	4.6	(1.5)	4.4	(2.0)
Undergone Surgery	2.8	(2.1)	3.0	(1.4)	2.6	(1.6)	4.1	(1.6)	5.8	(1.6)	4.6	(1.6)	4.5	(2.0)
Not Undergone Surgery	2.6	(2.0)	2.9	(1.5)	3.2	(1.7)	4.2	(1.7)	5.6	(1.7)	4.4	(1.5)	4.2	(2.1)
mJOA Upper Limb Function <3	2.9	(2.0)	2.1	(1.3)	2.5	(1.3)	3.7	(1.6)	6.0	(1.7)	4.4	(1.3)	4.2	(2.1)
mJOA Upper Limb Function >3	2.6	(2.0)	3.1	(1.5)	3.0	(1.7)	4.2	(1.7)	5.7	(1.7)	4.5	(1.6)	4.3	(2.1)
mJOA Lower Limb Function <4	2.8	(2.0)	2.9	(1.5)	2.4	(1.5)	4.1	(1.6)	5.7	(1.7)	4.6	(1.4)	4.3	(2.1)
mJOA Lower Limb Function >4	2.6	(2.1)	3.0	(1.5)	3.2	(1.7)	4.2	(1.7)	5.7	(1.7)	4.5	(1.6)	4.3	(2.0)
mJOA Upper Limb Sensation <2	2.7	(2.1)	2.6	(1.3)		(1.5)	4.2	(1.5)	5.9	(1.6)	4.5	(1.5)	4.2	(2.1)
mJOA Upper Limb Sensation >2	2.6	(2.0)	3.2	(1.6)	3.0	(1.8)	4.1	(1.8)	5.6	(1.7)	4.5	(1.6)	4.4	(2.1)
mJOA Bladder Function <2	2.6	(1.8)	2.8	(1.6)	2.5	(1.4)	3.6	(1.4)	6.0	(1.3)	4.7	(1.6)	4.7	(2.0)
mJOA Bladder Function >2	2.7	(2.1)	3.0	(1.5)	3.1	(1.7)	4.3	(1.7)	5.6	(1.7)	4.5	(1.5)	4.2	(2.1)
Length of Symptoms <3 years	2.9	(2.2)	3.0	(1.5)	3.1	(1.6)	4.2	(1.7)	5.6	(1.7)	4.6	(1.5)	4.1	(2.1)
Length of Symptoms >3 years	2.5	(1.9)	2.9	(1.4)	2.8	(1.7)	4.1	(1.7)	5.8	(1.6)	4.5	(1.5)	4.5	(2.1)
Best Limb Pain VAS <3	2.7	(2.1)	3.0	(1.5)	2.9	(1.7)	4.2	(1.7)	5.7	(1.7)	4.4	(1.5)	4.6	(2.0)
Best Limb Pain VAS >3	2.5	(2.0)	2.9	(1.5)	3.0	(1.6)	4.1	(1.6)	5.8	(1.6)	4.7	(1.6)	3.9	(2.2)

For peer review only

Supplementary Data 4: Questionnaire. The questions relevant to this study were developed, piloted and embedded within an ongoing initiative investigating patient reporting of DCM. The questions pertaining to the data points required for this study are presented, including their question number and options for selection. Question 35 about age was the only question where respondents were asked to type in a specific answer. The answer format was electronically validated to require an integer, prompting users to specify to the nearest year.

Questions generating data points required for this study	
5	How long have you suffered with cervical myelopathy?<br>0-1 year<br>1-3 years<br>3-10 years<br>10-25 years<br>>25 years
7	Have you undergone surgery for cervical myelopathy?<br>Yes<br>No
23	Currently, please indicate the intensity of the current, best and worst pain affecting your arms or legs over the past 24h on a scale of 0 (no pain) to 10 (worst pain imaginable)<br>0<br>1<br>2<br>3<br>4<br>5<br>6<br>7<br>8<br>9<br>10
29	How does cervical myelopathy affect your arms and hands? *Choose the statement that best fits: I am*<br>- Unable to move my hands<br>- Unable to eat with a spoon but am able to move my hands<br>- Unable to button my shirt but able to eat with a spoon<br>- Able to button my shirt with great difficulty<br>- Able to button my shirt with slight difficulty<br>- Not having any trouble using my hands.

For peer review only

30	How does cervical myelopathy affect your legs? *Choose the statement that best fits: I am*<br><br>- Completely unable to move legs at all and have no feeling in legs<br>- Having feeling in legs but not able to move them at all<br>- Able to move my legs but am unable to walk<br>- Able to walk on flat floor with a walking aid (cane or crutch)<br>- Able to walk up and/or downstairs with the aid of a handrail<br>- Able to walk up and/or downstairs without handrail but I notice moderate-to-significant lack of stability/feeling of imbalance when I walk<br>- Able to walk unaided (no crutches, canes, walker) with smooth reciprocation (ie, legs move smoothly) but I still notice mild lack of stability/feeling of imbalance when walking<br>- Able to walk without any problems of imbalance or instability
31	How does cervical myelopathy affect your arms and hands? *Choose the statement that best fits: I have*<br><br>- Complete loss of feeling in hands<br><br>- Severe loss of feeling, or have pain in my hands<br><br>- Mild loss of feeling in hands<br><br>- No loss of feeling in hands
32	How does cervical myelopathy affect your bladder? *Choose the statement that best fits: I am*<br><br>- Am completely unable to control urination<br><br>- Have marked difficulty controlling urination<br><br>- Have mild to moderate difficulty controlling urination<br><br>- No difficulty controlling urination
33	Effective medical research should target the needs of patients. The consequences of spinal cord injury can be classified into 7 different

For peer review only

	categories. In DCM, the patient priorities are not known.<br><br>For you as a patient, what are the research priorities for you (please rank where 1 is the most important and 7 is the least important)? What would you like researchers to focus on?<br><br>-Elimination of Pain<br>-Arm/Hand Function<br>-Walking Function<br>-Bladder/Bowel Function<br>-Sexual Function<br>-Upper Body/Trunk Function<br>-Normal Sensation
34	Are you male or female?<br><br>Male<br>Female
35	How old are you?

For peer review only

STROBE Statement

Checklist of items that should be included in reports of observational studies

Section/Topic	Item No	Recommendation	Reported on Page No
**Title and abstract**	1	(*a*) Indicate the study's design with a commonly used term in the title or the abstract	2
		(*b*) Provide in the abstract an informative and balanced summary of what was done and what was found	2
**Introduction**			
Background/rationale	2	Explain the scientific background and rationale for the investigation being reported	4-5
Objectives	3	State specific objectives, including any prespecified hypotheses	5
**Methods**			
Study design	4	Present key elements of study design early in the paper	6-7
Setting	5	Describe the setting, locations, and relevant dates, including periods of recruitment, exposure, follow-up, and data collection	6-7
Participants	6	(*a*) *Cohort study*—Give the eligibility criteria, and the sources and methods of selection of participants. Describe methods of follow-up<br>*Case-control study*—Give the eligibility criteria, and the sources and methods of case ascertainment and control selection. Give the rationale for the choice of cases and controls<br>*Cross-sectional study*—Give the eligibility criteria, and the sources and methods of selection of participants	6-7
		(*b*) *Cohort study*—For matched studies, give matching criteria and number of exposed and unexposed<br>*Case-control study*—For matched studies, give matching criteria and the number of controls per case	
Variables	7	Clearly define all outcomes, exposures, predictors, potential confounders, and effect modifiers. Give diagnostic criteria, if applicable	6-7
Data sources/measurement	8*	For each variable of interest, give sources of data and details of methods of assessment (measurement). Describe comparability of assessment methods if there is more than one group	6-7
Bias	9	Describe any efforts to address potential sources of bias	6-7
Study size	10	Explain how the study size was arrived at	6-7
Quantitative variables	11	Explain how quantitative variables were handled in the analyses. If applicable, describe which groupings were chosen and why	6-7
Statistical methods	12	(*a*) Describe all statistical methods, including those used to control for confounding	7
		(*b*) Describe any methods used to examine subgroups and interactions	7
		(*c*) Explain how missing data were addressed	7
		(*d*) *Cohort study*—If applicable, explain how loss to follow-up was addressed<br>*Case-control study*—If applicable, explain how matching of cases and controls was addressed<br>*Cross-sectional study*—If applicable, describe analytical methods taking account of sampling strategy	7
		(*e*) Describe any sensitivity analyses	

For peer review only - http://bmjopen.bmj.com/site/about/guidelines.xhtml

Section/Topic	Item No	Recommendation	Reported on Page No
**Results**			
Participants	13*	(a) Report numbers of individuals at each stage of study—eg numbers potentially eligible, examined for eligibility, confirmed eligible, included in the study, completing follow-up, and analysed	8
		(b) Give reasons for non-participation at each stage	N/A
		(c) Consider use of a flow diagram	N/A
Descriptive data	14*	(a) Give characteristics of study participants (eg demographic, clinical, social) and information on exposures and potential confounders	8
		(b) Indicate number of participants with missing data for each variable of interest	Supplementary Data 2
		(c) *Cohort study*—Summarise follow-up time (eg, average and total amount)	N/A
Outcome data	15*	*Cohort study*—Report numbers of outcome events or summary measures over time	N/A
		Case-control study—Report numbers in each exposure category, or summary measures of exposure	N/A
		Cross-sectional study—Report numbers of outcome events or summary measures	12-13
Main results	16	(*a*) Give unadjusted estimates and, if applicable, confounder-adjusted estimates and their precision (eg, 95% confidence interval). Make clear which confounders were adjusted for and why they were included	8
		(*b*) Report category boundaries when continuous variables were categorized	Supplementary Data 1,3 & 4
		(*c*) If relevant, consider translating estimates of relative risk into absolute risk for a meaningful time period	N/A
Other analyses	17	Report other analyses done—eg analyses of subgroups and interactions, and sensitivity analyses	8-9
**Discussion**			
Key results	18	Summarise key results with reference to study objectives	10
Limitations	19	Discuss limitations of the study, taking into account sources of potential bias or imprecision. Discuss both direction and magnitude of any potential bias	11
Interpretation	20	Give a cautious overall interpretation of results considering objectives, limitations, multiplicity of analyses, results from similar studies, and other relevant evidence	10-11
Generalisability	21	Discuss the generalisability (external validity) of the study results	10-11
**Other Information**			
Funding	22	Give the source of funding and the role of the funders for the present study and, if applicable, for the original study on which the present article is based	1

Give information separately for cases and controls in case-control studies and, if applicable, for exposed and unexposed groups in cohort and cross-sectional studies.

For peer review only - http://bmjopen.bmj.com/site/about/guidelines.xhtml

Note: An Explanation and Elaboration article discusses each checklist item and gives methodological background and published examples of transparent reporting. The STROBE checklist is best used in conjunction with this article (freely available on the Web sites of PLoS Medicine at http://www.plosmedicine.org/, Annals of Internal Medicine at http://www.annals.org/, and Epidemiology at http://www.epidem.com/). Information on the STROBE Initiative is available at www.strobe-statement.org.

For peer review only

BMJ Open

Recovery priorities in degenerative cervical myelopathy: a cross-sectional survey of an international, online community of patients

Journal:	*BMJ Open*
Manuscript ID	bmjopen-2019-031486.R2
Article Type:	Research
Date Submitted by the Author:	11-Aug-2019
Complete List of Authors:	Davies, Benjamin; University of Cambridge, Department of Clinical Neurosurgery<br>Mowforth, Oliver; University of Cambridge, Division of Neurosurgery, Department of Clinical Neurosciences<br>Sadler, Iwan; Myelopathy.org<br>Aarabi, Bizhan; University of Maryland Baltimore, Division of Neurosurgery<br>Kwon, Brian; University of British Columbia<br>Kurpad, Shekar; Medical College of Wisconsin, Department of Neurosurgery<br>Harrop, James; Toronto Western Hospital,<br>Wilson, Jefferson R.; University of Toronto<br>Grossman, Robert; Houston Methodist Hospital<br>Fehlings, Michael G.; Toronto Western Hospital,<br>Kotter, Mark; University of Cambridge, Department of Clinical Neurosurgery; Ann McLaren Laboratory of Regenerative Medicine, Department of Clinical Neurosciences
**Primary Subject Heading**:	Patient-centred medicine
Secondary Subject Heading:	Neurology
Keywords:	Spine < ORTHOPAEDIC & TRAUMA SURGERY, NEUROSURGERY, Neurosurgery < SURGERY, Neurological pain < NEUROLOGY

SCHOLARONE™
Manuscripts

Recovery priorities in degenerative cervical myelopathy: a cross-sectional survey of an international, online community of patients

Benjamin M. Davies M.R.C.S B.Sc[1], Oliver D. Mowforth BA. MB. BChir.[1], Iwan Sadler[2], Bizhan Aarabi MD[3], Brian K. Kwon M.D. Ph.D F.R.C.S.C[4], Shekar N. Kurpad M.D Ph.D[5], James Harrop M.D.[6] Jefferson R. Wilson M.D. Ph.D[7], Robert Grossman M.D.[8], Michael G. Fehlings M.D. Ph.D F.R.C.S.C[9] , Mark R.N. Kotter M.D. M.Phil. Ph.D.[1,10]

1) Academic Neurosurgery Unit, Department of Clinical Neurosurgery, University of Cambridge, Cambridge, UK
2) DCM Sufferer and Director of Myelopathy Support, Myelopathy.org, Cambridge, UK
3) Division of Neurosurgery, Shock Trauma, University of Maryland, Baltimore, Maryland, USA
4) Division of Spine Surgery, Vancouver General Hospital, University of British Columbia, Vancouver, British Columbia, Canada
5) Department of Neurosurgery, Medical College of Wisconsin, Milwaukee, Wisconsin, USA
6) Division of Neurosurgery, Thomas Jefferson University Hospital, Philadelphia, Pennsylvania, USA
7) Division of Neurosurgery, Department of Surgery, University of Toronto, Toronto, Ontario, Canada
8) Division of Neurosurgery, Houston Methodist Hospital , Houston, Texas, USA
9) Division of Neurosurgery and Spine Program, Toronto Western Hospital, University Health Network, University of Toronto, Toronto, Ontario, Canada
10) Wellcome Trust & MRC Cambridge Stem Cell Institute, UK

Running title: Pain is the recovery priority in degenerative cervical myelopathy

Key words: cervical; myelopathy; spondylosis; spondylotic; stenosis; disc herniation; ossification posterior longitudinal ligament; degeneration; disability; recovery; questionnaire; priority setting; patient and public involvement; research wastage.

Main text word count*:* 2710

Reference count: 35

Funding: Research in the senior author's laboratory is supported by a core support grant from the Wellcome Trust and MRC to the Wellcome Trust-Medical Research Council Cambridge Stem Cell Institute. MRNK is supported by a NIHR Clinician Scientist Award. MGF is supported by the Halbert Chair in Neural Repair and Regeneration. Partial support for this work was obtained from the AOSpine Knowledge Forum in Spinal Cord Injury.
The authors also acknowledge the AOSpine for their support of travel and meetings costs.
The NIHR HTC, from the Brain Injury Medical Technology Cooperative, provided the funding for Google Adwords survey advertising.

Disclaimer: This report is independent research arising from a Clinician Scientist Award, CS-2015-15-023, supported by the National Institute for Health Research. The views expressed in this publication are those of the authors and not necessarily those of the NHS, the National Institute for Health Research or the Department of Health and Social Care.

Correspondence to:
Dr Mark Kotter
Anne McLaren Laboratory
Department of Clinical Neurosciences
WT MRC Cambridge Stem Cell Institute
West Forvie Building, Forvie Site
University of Cambridge
CB2 0SZ
Email: mrk25@cam.ac.uk
Telephone: +44 1223 763366
Fax: +44 1223 763350

For peer review only

Abstract

Objectives

To establish the recovery priorities of individuals suffering with degenerative cervical myelopathy (DCM).

Design

A cross-sectional, observational study.

Setting

Patients from across the world with a diagnosis of DCM accessed the survey over an 18-month period on Myelopathy.org, an international myelopathy charity.

Participants

481 individuals suffering from DCM completed the online survey fully.

Main outcome measures

Functional recovery domains were established through qualitative interviews and a consensus process. Individuals were asked about their disease characteristics, including limb pain (visual analogue scale) and functional disability (patient derived - modified Japanese Orthopaedic Association score). Individuals ranked recovery domains (arm and hand function, walking, upper body/trunk function, sexual function, elimination of pain, sensation and bladder/bowel function) in order of priority. Priorities were analysed as the modal first priority and mean ranking. The influence of demographics on selection was analysed, with significance $p<0.05$.

Results

Of 659 survey responses obtained, 481 were complete. Overall, pain was the most popular recovery priority (39.9%) of respondents, followed by walking (20.2%), sensation (11.9%) and arm and hand function (11.5%). Sexual function (5.7%), bladder and bowel (3.7%) or trunk function (3.5%) were chosen less frequently. When considering the average ranking of symptoms, whilst pain remained the priority (2.6±2.0), this was closely followed by walking (2.9±1.7) and arm/hand function (3.0 ±1.4). Sensation ranked much lower (4.3±2.1). With respect to disease characteristics, overall pain remained the recovery priority, with the exception of patients with greater walking impairment ($p<0.005$) who prioritised walking, even amongst patients with lower pain scores.

Conclusions

This is the first study investigating patient priorities in DCM. The patient priorities reported provide an important framework for future research and will help ensure that it is aligned with patient needs.

For peer review only

Strengths

- **This is the largest study of patient perspective in DCM to date and the first to consider patient recovery priorities**
- **This study is unique in reporting on both surgical and non-surgical DCM patients.**
- **This study includes a broad demographic representation of patients from across the globe and includes subgroup analysis.**

Limitations

- **This is an open-access, internet-based survey, a methodology which can lead to a sampling bias.**
- **Efforts to mitigate against sampling bias, alongside reassuring sub-group analysis suggest this risk is low.**

For peer review only

Introduction

Degenerative cervical myelopathy [DCM] has been coined as an umbrella term for degenerative and congenital or acquired conditions of the cervical spine, such as spondylosis or ossification of the posterior longitudinal ligament (OPLL), which lead to symptomatic cord compression.[1] With an estimated prevalence of up to 5% in individuals above 40 years old,[2],[3] DCM is the most common cause of spinal cord dysfunction worldwide.[1] Given its degenerative aetiology and the rising age of the population, this incidence is expected to rise.[4]

The cervical spinal cord acts as a processor and conduit of information between the brain and the periphery. Its injury can therefore give rise to a range of possible symptoms.[1] These include pain, paraesthesia, weakness, unsteadiness, frequent falls, bladder or bowel dysfunction and impotence in men.[5] At early stages, individual symptoms may occur in isolation, but more typically occur in combination, especially as the disease advances.

At present, decompressive surgery is the only evidence-based treatment for DCM.[6]. Surgical decompression is able to halt the progression of symptoms and offer limited, albeit clinically relevant[7] improvements across a range of domains.[8],[9] However, due to the limited intrinsic capacity for the spinal cord to repair, most patients do not make a full recovery, and instead suffer lifelong disabilities.[9] As a consequence, unemployment and/or dependency is prevalent amongst individuals with DCM.[4],[10],[11] Moreover, a recent study has identified that DCM severely impacts quality of life with recorded SF-36, patient reported outcome scores amongst the lowest of all chronic disease.[12] Improving recovery is therefore a major unmet clinical need in DCM.[13]

Medical research is primarily designed by health care professionals. This bears the risk of not taking into account actual patient needs. The concept of 'research wastage' has emerged to depict healthcare research that does not yield actual or potential clinical benefit. In the 2014 *Lancet* series, Chalmers et al. estimated that as much as 85% of the US$240 billion expended on health research in 2010 was wasted and an important contributing factor was the misalignment of patient and clinician research objectives.[14],[15] As a consequence, several research funding bodies now advocate the involvement of patients in the design and conduct of research. This has demonstrable beneficial impact.[16] Patient and public involvement (PPI) plays a particularly important role in the National Institute for Health Research (NIHR).[17] In addition to participation and engagement in the research process, the involvement of patients in identifying relevant research topics and their prioritisation is particularly encouraged. Organisations such as the James Lind Alliance have

For peer review only

successfully brought together patients, professionals and industry in order to set research priorities, e.g. for spinal cord injury.[18] However, the research priorities for individuals suffering from DCM have not yet been assessed.

A recent systematic review of DCM research demonstrated a heavy focus on surgical technique.[19],[20] However, the research needs of patients with DCM and their priorities remain unknown. Moreover, as part of a core-outcomes initiative (REsearch Objectives and COmmon Date Elements in DCM [RECODE-DCM]) we have identified that outcome domains are not consistently reported in current clinical research.[19]

In this study we sought to establish the recovery needs and priorities of individuals suffering from DCM. This will help to determine the outcome assessments that should be included in clinical research and to better direct future research.

For peer review only

Methods

Reporting adheres to the EQUATOR Network STROBE checklist[21].

Survey design

Individuals with DCM and their caregivers were invited to attend the Myelopathy.org Patient and Public Involvement day, hosted at the University of Cambridge and captured by Cambridge TV in their documentary.[22] Myelopathy.org is an international, charitable organisation for individuals affected by or working with DCM. As part of the event, qualitative interviews (N=9) were used to establish relevant functional domains that affected quality of life of individuals with DCM. These were found to resemble domains previously reported by Anderson et al. (2004), who conducted a survey amongst patients with traumatic spinal cord injury asking them to rank seven domains of spinal cord function in order of priority for recovery.[23] Using this as a template but broadening 'upper body/trunk strength and balance' to upper body/trunk function, the following recovery domains were agreed by participants: elimination of pain, arm and hand function, walking, sexual function, upper body/trunk function, sensation and bladder/bowel function. For brevity, in this article they are referred to as arm/hand, walking, sexual function, pain, sensation, trunk and bladder/bowel.

These questions were embedded into an existing electronic survey initiative, developed using Survey Monkey (California, USA) and following the Checklist for Reporting Results of Internet E-Surveys (CHERRIES),[24] investigating patient reporting of DCM. This iteration was piloted by the lead investigators and a selection of individuals with DCM. Ethical approval was granted by the University of Cambridge. Study objectives were outlined on the initial page, including details of the host organization and estimated time required to complete the survey. This acted as the electronic consent, with continuation into the survey as agreement. Respondents were also presented with a description of DCM, including relevant synonyms, and required to confirm they suffered with the condition.

Respondents were asked to rank recovery domains in order of priority and provide details about their DCM. DCM characteristics included age, gender, history of surgery, best daily limb pain score (using a visual analogue scale), duration of symptoms and disease severity as measured using the self-reported, patient-derived, modified Japanese Orthopaedic Association [**P**-mJOA]. [25] The modified Japanese Orthopaedic scale [mJOA] is amongst the most commonly utilised assessments of disease severity[19],[20] and is fully validated.[26] It is a composite score based on upper limb function, lower limb function, upper limb sensation and bladder function. The score is valid for analysis in its entirety or per domain. Originally developed as an investigator administered tool, it has recently been

For peer review only - http://bmjopen.bmj.com/site/about/guidelines.xhtml

adapted and validated for use by patients. [25] All questions were mandatory, but respondents were not required to rank every recovery domain, on the basis that some domains may not be a priority for them. The sequence of questions and order of responses was not altered from respondent to respondent.

Survey administration

The survey was accessed via a landing page on Myelopathy.org, allowing assessment of response rates using Google Analytics (California, USA). Individuals with DCM were recruited over an eighteen-month period. The recruitment process has been described in detail previously[27] but in short, the survey was advertised using Google Adwords (California, USA) and through Myelopathy.org and its social media outlets. The survey was voluntary and internet protocol addresses were used to prevent users submitting multiple responses. A missing data analysis was conducted between complete and incomplete survey responses to consider if particular subgroups were more likely to terminate early. Complete responders were defined as having provided answers for all aforementioned variables.

Analysis

Research priorities are presented using summary statistics, including average ranking and overall proportion of patients per domain. Domains which were not ranked by a respondent were omitted from these scores. For subgroup analysis, variables were dichotomised and thresholds were chosen based on the graphical distribution of responses and sample sizes. Categorical variables were compared using the Chi-Squared test. For continuous variables, the Shapiro-Wilks test was used to assess for parametric distribution of data sets. The Mann-Whitney U test was then used to compare the means of non-parametric distributions whilst a two-tailed T-test used to compare the means of parametric distributions. Pearson's correlations were performed to assess between group differences in characteristics, which could have influenced sub-group analysis. Significance was set at p <0.05.

Patient and Public Involvement

Patients were involved in the design, development, recruitment and conduct of this study. At a patient and public involvement day hosted at the University of Cambridge, a focus group of DCM patients evaluated and confirmed the recovery domains in DCM. DCM patients were used to pilot the subsequent survey, including optimising its design to reduce the time taken to complete and clarify questions. The online survey for the study was hosted on Myelopathy.org, an international DCM charity run largely by DCM patients. Patients were therefore active in disseminating the survey via online DCM support groups, including

For peer review only

Myelopathy Support, led by Iwan Sadler, patient and co-author. Patients who were involved in preparation of the manuscript are amongst the authors. In addition, all patients who participated in the research are recognised in the acknowledgement statement. DCM patients are involved in plans to disseminate this research to the patient community, including blog articles on Myelopathy.org, posts in online patient support groups and presence at spinal conferences in the United Kingdom

For peer review only

Results

Respondents

The survey was uniquely accessed 1463 times, with 659 visitors entering the survey (participation rate of 33%). A total of 481 responses contained complete data (completion rate 73%). A missing data analysis was conducted comparing incomplete and complete responses. Patients who completed the survey in full were more likely to have undergone surgery (p = 0.04), otherwise there was no statistical difference within variables of interest (Supplementary Data 1). Only complete responses were analysed in the present study. Of these responses domains were ranked more than 80% of the time: pain (400, 83%), sensation (428, 89%), walking (396, 82%), arm and hand (393, 82%), sexual (388, 81%), bladder and bowel (399, 83%) and trunk function (407, 85%).

On average respondents were more likely to be female (341, 70%) and suffer with moderate myelopathy (11.9 ±3.0) for between 3 and 10 years (181, 31%). Around half of patients (221, 46%) had undergone surgery. Overall respondent demographics are summarised in Table 1. Considering group differences, patients who had suffered from the disease for longer were more likely to have undergone surgery (p <0.01) and have worse myelopathy (-0.22, p <0.005). They were also more likely to suffer more pain (-0.14, p <0.01). Average pain scores were 3.1 (±2.4) for patients suffering with the disease for less than a year, rising to 4.5 (±3.0) for patients suffering for at least 10 years. There was no relationship between severity of myelopathy and pain scores (-0.04, p = 0.36). Between group differences are summarised in Supplementary Data 2.

Ranking of spinal cord dysfunction domains

Overall, pain was the most popular number one ranked recovery domain, chosen by 39.9% of respondents. This was followed by walking (20.2%), sensation (11.9%) and arm and hand function (11.5%). Sexual function (5.7%), bladder and bowel (3.7%) or trunk function (3.5%) were chosen less frequently. When considering the average ranking of symptoms, whilst pain remained the priority (2.6±2.0), this was closely followed by walking (2.9±1.7) and arm/hand function (3.0 ±1.4) (Figure 1). Sensation ranked lower (4.3±2.1).

Impact of baseline characteristics on ranking of spinal cord dysfunction domains

Respondents who had undergone surgery were more likely to prioritise walking (p < 0.005) and trunk function (p = 0.03), whereas patients who had not yet undergone surgery were more likely to prioritise upper limb function (p < 0.05) (Figure 2). Patients with poor upper

For peer review only

limb or lower limb function were more likely to prioritise arm/hand recovery ($p < 0.005$) and walking ($p < 0.005$) respectively (Figure 2). Overall, pain remained the priority, with the exception of patients with greater walking impairment ($p < 0.005$), even amongst patients with lower pain scores (Figure 2).

When considering the average rankings pain, arm/hand function and walking remained the top three recovery priorities (Figure 3). However, amongst the subcategories, the order of these priorities differed slightly (Supplementary Data 3). Patients who were male, or who had undergone surgery, or who had greater lower limb or bladder functional disability, prioritised recovery of walking, over pain and arm/hand function; patients with greater upper limb function or sensory disability prioritised recovery of arm/hand function over pain and walking.

When overall P-mJOA scores were considered to evaluate mild, moderate and severe patients,[6] no variation was seen in modal or average ranked priorities.

For peer review only

Discussion

This is the first study to systematically survey functional domains relevant to DCM and to ask patients to rank them in order of importance to their quality of life. The established priorities are likely to reflect symptom prevalence and their impact on day to day life.[23] The analysis of 481 completed answers demonstrated that pain, arm/hand function and walking emerge as the most important spinal cord dysfunction domains. Although based on averaged rankings, there were some subtle differences in ordering of these three domains. With the exception of patients with significant gait impairment, elimination of pain was the recovery priority independent of baseline characteristic.

These findings are surprising: functional disability (specifically recovery of arm/hand and walking function) has been and continues to be a focus for researchers, typically in response to surgery,[8] but more recently with a shift towards enhancing post-surgical recovery.[13],[27] In contrast, pain is not widely recognised as an important relevant domain. Our recent review of outcome reporting in DCM clinical trials demonstrated that the overwhelming majority of studies (90%) reported outcomes related to function, but only 27% of studies reported outcomes related to pain,[19] despite the fact that pain is a well-recognised feature of DCM,[5] which often improves following surgery.[11] The present findings highlight the fact that systematic research of patient needs is sorely lacking in DCM. A possible explanation for this discrepancy is that surgeons, who play a significant role in the management of DCM and predominate this research field, remain biased towards functional domains because pain is not a recognised indication for surgery in DCM.[6]

The priorities established in the present study differ from those of individuals suffering from spinal cord injury. Although pain is amongst the most prevalent symptoms of traumatic spinal cord injury,[28],[29] the “elimination of chronic pain” was considered to be a relatively low priority amongst those surveyed in Anderson’s study[23] and a similar study by Kwon et al.[30] that focused on the priorities for SCI recovery after novel treatments (e.g. stem cells). Instead, quadriplegics prioritised arm/hand function, whilst paraplegics sexual and bladder/bowel function. These differences relate to their specific significance for patient independence and quality of life.

In DCM, the symptom burden is less well-described[31],[32] and the relationship between symptom burden or their significance with respect to quality of life in DCM has not been investigated. However, it would seem likely a similar relationship exists.

For peer review only

Limitations

Following recommendation by the James Lind Alliance, which was founded to support priority setting in research,[33],[34] the present survey was conducted online, as previously described through a DCM charity, Myelopathy.org.[27] Respondents belonged to a self-selecting group of individuals who were asked to confirm they had a diagnosis of DCM by a medical professional, after being presented with an explanation of the disease for verification purposes. It is possible that some respondents did not have DCM. Reassuringly, respondent demographics were comparable to those of leading prospective surgical studies, with the exception of gender which was not shown to influence patient priorities[8],[9] (Supplementary Data 1). This likely reflects the recognised popularity of online health communities amongst females. There are no such comparable series for non-surgical cohorts, but their inclusion provides a further valuable perspective.

The survey questions were not randomly sorted and therefore each respondent answered identical surveys with spinal cord function domains presented in the same order. The last domain assessed was sensation. In keeping with it being the most prevalent DCM symptom,[32] it featured most frequently in the responses, indicating that the order of domains was unlikely to have influenced the rankings. Moreover, answers to demographic questions, which followed the ranking of priorities on the survey, were required to define a complete response in order to be included in the present analysis. Priorities therefore were not influenced by incomplete answers.

Following the qualitative development work and the previous experience of Anderson et al., 2004, the pain domain was kept non-specific, asking patients to rank 'elimination of pain' as a recovery priority (Supplementary Data 4). In contrast however, the pain assessment focused on limb pain, which is classically felt to represent DCM-related pain.[5] Whilst this does not limit the implications of our findings as whole, their interpretation will require a better characterisation of pain in DCM in order to focus research appropriately as other pain foci are reported.[35]

Conclusion

The priorities reported in the present study identify functional domains that are relevant to the quality of life of DCM patients. They provide an important framework for future research

For peer review only

and will serve as a valuable reference for the development of a core outcome set relevant to studies in DCM.

For peer review only

Table

Table 1: Summary of respondent demographics.

Respondent Demographics	
Age (Mean ± SD)	53.6 (9.8)
Male Gender (%)	140 (29)
Undergone Surgery (%)	221 (46)
Length of Symptoms (%)	
0 to 1 year	72 (15)
1 to 3 years	140 (29)
3 to 10 years	181 (38)
10 to 25 years	74 (15)
25+ years	14 (3)
P-mJOA (Mean +SD)	
Upper Limb Function	3.6 (1.0)
Walking	4.4 (1.5)
Upper Limb Sensation	1.7 (0.7)
Bladder Function	2.2 (1.0)
Total	11.9 (3.0)
VAS Limb Pain (Mean ± SD)	3.1 (2.6)

For peer review only

Figure Legends

Figure 1: Overall recovery priorities. The bar chart represents the first choice of patients and the line graph the average ranking for each domain (where the top ranking is 1). Pain was the overall first choice priority of patients, although when priority rankings were averaged, this was closely followed by walking and arm/hand function.

Figure 2: Impact of baseline characteristics on first choice recovery priority. The bar chart represents the first choice of patients. Significant between group differences are denoted by the * symbol. For simplicity, groups were dichotomised as follows: Duration <3years, P-mJOA upper limb function <3, P-mJOA lower limb function <4 or feeling <2, P-mJOA bladder/bowel function <2 and VAS limb pain <3. Those who had undergone surgery were more likely to choose trunk function ($p=0.03$) or walking function ($p<0.005$), whereas those who had not yet undergone surgery were more likely to choose arm/hand function ($p<0.05$). Equally patients with more impairment of walking ($p<0.005$) or arm/hand function ($p<0.005$) were more likely to prioritise these domains. Pain remained the priority even in patients reporting less pain.

Figure 3: Impact of baseline characteristics on recovery priority average rankings. The scatter plot represents the mean ranking for each subgroup investigated. The blue line represents the overall average. Despite some discrepancies between subgroups, pain, arm/hand and walking function were consistently the top three priorities for patients. Bladder/bowel function was not a recovery priority.

For peer review only

Acknowledgements

The authors are grateful to all DCM patients who participated in this research.

Declaration Statement:

JH reports being a Medical Advisor for Depuy Synthes and Ethicon, being an Educational Speaker at Globus Medical and research funding from AO Spine. MGF reports consulting for Fortuna Fix. MRK declares a grant from the National Institute for Health Research, travel support from AO Spine and is founder of Myelopathy.org, the first charity for patients with cervical myelopathy. The remaining authors have nothing to declare.

Author Contributions Statement:

BD, OM, IS, BA, BK, SK, JH, JW, RG, MF and MK were involved in the interpretation, drafting and final approval of the manuscript. Additionally, authors BD, IS and MK were involved in the conception, design and acquisition of data for this study, whilst authors BD and OM conducted the data analysis.

Data Availability Statement:

All relevant data to the study are included in the article or uploaded as supplementary information. No additional data are available.

For peer review only

For peer review only

References:

1 Davies BM, Mowforth OD, Smith EK, *et al.* Degenerative cervical myelopathy. *BMJ* 2018;**360**:k186. doi:10.1136/bmj.k186

2 Kovalova I, Kerkovsky M, Kadanka Z, *et al.* Prevalence and Imaging Characteristics of Nonmyelopathic and Myelopathic Spondylotic Cervical Cord Compression. *Spine* 2016;**41**:1908–16. doi:10.1097/BRS.0000000000001842

3 Kadanka Z, Adamova B, Kerkovsky M, *et al.* Predictors of symptomatic myelopathy in degenerative cervical spinal cord compression. *Brain Behav* 2017;**7**. doi:10.1002/brb3.797

4 Nouri A, Tetreault L, Singh A, *et al.* Degenerative Cervical Myelopathy: Epidemiology, Genetics, and Pathogenesis. *Spine* 2015;**40**:E675-693. doi:10.1097/BRS.0000000000000913

5 Tracy JA, Bartleson JD. Cervical spondylotic myelopathy. *The Neurologist* 2010;**16**:176–87. doi:10.1097/NRL.0b013e3181da3a29

6 Fehlings MG, Tetreault LA, Riew KD, *et al.* A Clinical Practice Guideline for the Management of Patients With Degenerative Cervical Myelopathy: Recommendations for Patients With Mild, Moderate, and Severe Disease and Nonmyelopathic Patients With Evidence of Cord Compression. *Glob Spine J* 2017;**7**:70S-83S. doi:10.1177/2192568217701914

7 Tetreault L, Wilson JR, Kotter MRN, *et al.* Predicting the minimum clinically important difference in patients undergoing surgery for the treatment of degenerative cervical myelopathy. *Neurosurg Focus* 2016;**40**:E14. doi:10.3171/2016.3.FOCUS1665

8 Fehlings MG, Wilson JR, Kopjar B, *et al.* Efficacy and safety of surgical decompression in patients with cervical spondylotic myelopathy: results of the AOSpine North America prospective multi-center study. *J Bone Joint Surg Am* 2013;**95**:1651–8. doi:10.2106/JBJS.L.00589

9 Fehlings MG, Ibrahim A, Tetreault L, *et al.* A global perspective on the outcomes of surgical decompression in patients with cervical spondylotic myelopathy: results from the prospective multicenter AOSpine international study on 479 patients. *Spine* 2015;**40**:1322–8. doi:10.1097/BRS.0000000000000988

10 Al-Tamimi YZ, Guilfoyle M, Seeley H, *et al.* Measurement of long-term outcome in patients with cervical spondylotic myelopathy treated surgically. *Eur Spine J Off Publ Eur Spine Soc Eur Spinal Deform Soc Eur Sect Cerv Spine Res Soc* 2013;**22**:2552–7. doi:10.1007/s00586-013-2965-4

11 Fehlings MG, Tetreault LA, Kurpad S, *et al.* Change in Functional Impairment, Disability, and Quality of Life Following Operative Treatment for Degenerative Cervical Myelopathy: A Systematic Review and Meta-Analysis. *Glob Spine J* 2017;**7**:53S-69S. doi:10.1177/2192568217710137

12 Oh T, Lafage R, Lafage V, *et al.* Comparing Quality of Life in Cervical Spondylotic Myelopathy with Other Chronic Debilitating Diseases Using the Short Form Survey 36-Health Survey. *World Neurosurg* 2017;**106**:699–706. doi:10.1016/j.wneu.2016.12.124

For peer review only

13 Fehlings MG, Wilson JR, Karadimas SK, *et al.* Clinical evaluation of a neuroprotective drug in patients with cervical spondylotic myelopathy undergoing surgical treatment: design and rationale for the CSM-Protect trial. *Spine* 2013;**38**:S68-75. doi:10.1097/BRS.0b013e3182a7e9b0

14 Chalmers I, Glasziou P. Avoidable waste in the production and reporting of research evidence. *Lancet Lond Engl* 2009;**374**:86–9. doi:10.1016/S0140-6736(09)60329-9

15 Chalmers I, Bracken MB, Djulbegovic B, *et al.* How to increase value and reduce waste when research priorities are set. *Lancet Lond Engl* 2014;**383**:156–65. doi:10.1016/S0140-6736(13)62229-1

16 Brett J, Staniszewska S, Mockford C, *et al.* Mapping the impact of patient and public involvement on health and social care research: a systematic review. *Health Expect Int J Public Particip Health Care Health Policy* 2014;**17**:637–50. doi:10.1111/j.1369-7625.2012.00795.x

17 National Institute for Health Research. Patient and Public Involvement. www.nihr.ac.uk. https://www.nihr.ac.uk/patients-and-public/ (accessed Feb 16, 2018).

18 van Middendorp JJ, Allison HC, Ahuja S, *et al.* Top ten research priorities for spinal cord injury: the methodology and results of a British priority setting partnership. *Spinal Cord* 2016;**54**:341–6. doi:10.1038/sc.2015.199

19 Davies BM, McHugh M, Elgheriani A, *et al.* Reported Outcome Measures in Degenerative Cervical Myelopathy: A Systematic Review. *PloS One* 2016;**11**:e0157263. doi:10.1371/journal.pone.0157263

20 Davies BM, McHugh M, Elgheriani A, *et al.* The reporting of study and population characteristics in degenerative cervical myelopathy: A systematic review. *PloS One* 2017;**12**:e0172564. doi:10.1371/journal.pone.0172564

21 Elm E von, Altman DG, Egger M, *et al.* Strengthening the reporting of observational studies in epidemiology (STROBE) statement: guidelines for reporting observational studies. *BMJ* 2007;**335**:806–8. doi:10.1136/bmj.39335.541782.AD

22 Roberts E. Elemental Ideas: Cervical Spondylotic Myelopathy. http://www.myelopathy.org/the-csm-documentary.html.

23 Anderson KD. Targeting recovery: priorities of the spinal cord-injured population. *J Neurotrauma* 2004;**21**:1371–83. doi:10.1089/neu.2004.21.1371

24 Eysenbach G. Improving the Quality of Web Surveys: The Checklist for Reporting Results of Internet E-Surveys (CHERRIES). *J Med Internet Res* 2004;**6**. doi:10.2196/jmir.6.3.e34

25 Rhee JM, Shi WJ, Cyriac M, *et al.* The P-mJOA: A Patient-derived, Self-reported Outcome Instrument for Evaluating Cervical Myelopathy: Comparison with the mJOA. *Clin Spine Surg* 2018;**31**:E115–20. doi:10.1097/BSD.0000000000000591

26 Kopjar B, Tetreault L, Kalsi-Ryan S, *et al.* Psychometric properties of the modified Japanese Orthopaedic Association scale in patients with cervical spondylotic myelopathy. *Spine* 2015;**40**:E23-28. doi:10.1097/BRS.0000000000000648

For peer review only

27 Davies B, Kotter M. Lessons From Recruitment to an Internet-Based Survey for Degenerative Cervical Myelopathy: Comparison of Free and Fee-Based Methods. *JMIR Res Protoc* 2018;**7**:e18. doi:10.2196/resprot.6567

28 Dijkers M, Bryce T, Zanca J. Prevalence of chronic pain after traumatic spinal cord injury: a systematic review. *J Rehabil Res Dev* 2009;**46**:13–29.

29 Siddall PJ, Loeser JD. Pain following spinal cord injury. *Spinal Cord* 2001;**39**:63–73.

30 Kwon BK, Ghag A, Dvorak MF, *et al.* Expectations of benefit and tolerance to risk of individuals with spinal cord injury regarding potential participation in clinical trials. *J Neurotrauma* 2012;**29**:2727–37. doi:10.1089/neu.2012.2550

31 Zhang RJ, Shen CL, Zhang JX, *et al.* Clinical features and surgical outcomes of cervical spondylotic myelopathy in patients of different ages: a retrospective study. *Spinal Cord* 2018;**56**:7–13. doi:10.1038/sc.2017.91

32 Nouri A, Tetreault L, Dalzell K, *et al.* The Relationship Between Preoperative Clinical Presentation and Quantitative Magnetic Resonance Imaging Features in Patients With Degenerative Cervical Myelopathy. *Neurosurgery* 2017;**80**:121–8. doi:10.1227/NEU.0000000000001420

33 Petit-Zeman S, Firkins L, Scadding JW. The James Lind Alliance: tackling research mismatches. *Lancet Lond Engl* 2010;**376**:667–9. doi:10.1016/S0140-6736(10)60712-X

34 Finer S, Robb P, Cowan K, *et al.* Top ten research priorities for type 2 diabetes: results from the Diabetes UK-James Lind Alliance Priority Setting Partnership. *Lancet Diabetes Endocrinol* 2017;**5**:935–6. doi:10.1016/S2213-8587(17)30324-8

35 Schrot RJ, Mathew JS, Li Y, *et al.* Headache relief after anterior cervical discectomy: post hoc analysis of a randomized investigational device exemption trial: clinical article. *J Neurosurg Spine* 2014;**21**:217–22. doi:10.3171/2014.4.SPINE13669

For peer review only

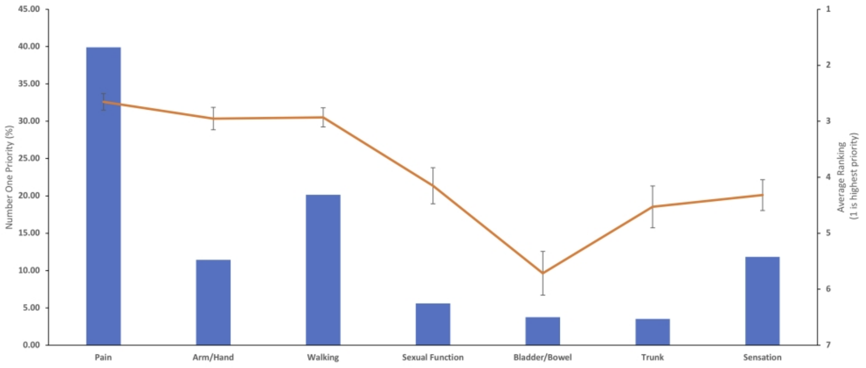

Figure 1: Overall recovery priorities. The bar chart represents the first choice of patients and the line graph the average ranking for each domain (where the top ranking is 1). Pain was the overall first choice priority of patients, although when priority rankings were averaged, this was closely followed by walking and arm/hand function.

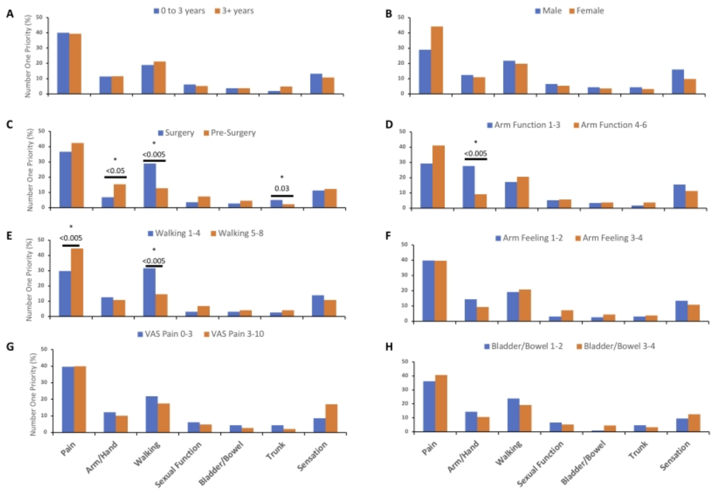

Figure 2: Impact of baseline characteristics on first choice recovery priority. The bar chart represents the first choice of patients. Significant between group differences are denoted by the * symbol. For simplicity, groups were dichotomised as follows: Duration <3years, mJOA upper limb function <3, mJOA lower limb function <4 or feeling <2, mJOA bladder/bowel function <2 and VAS limb pain <3. Those who had undergone surgery were more likely to choose trunk function (p=0.03) or walking function (p<0.005), whereas those who had not yet undergone surgery were more likely to choose arm/hand function (p<0.05). Equally patients with more impairment of walking (p<0.005) or arm/hand function (p<0.005) were more likely to prioritise these domains. Pain remained the priority even in patients reporting less pain.

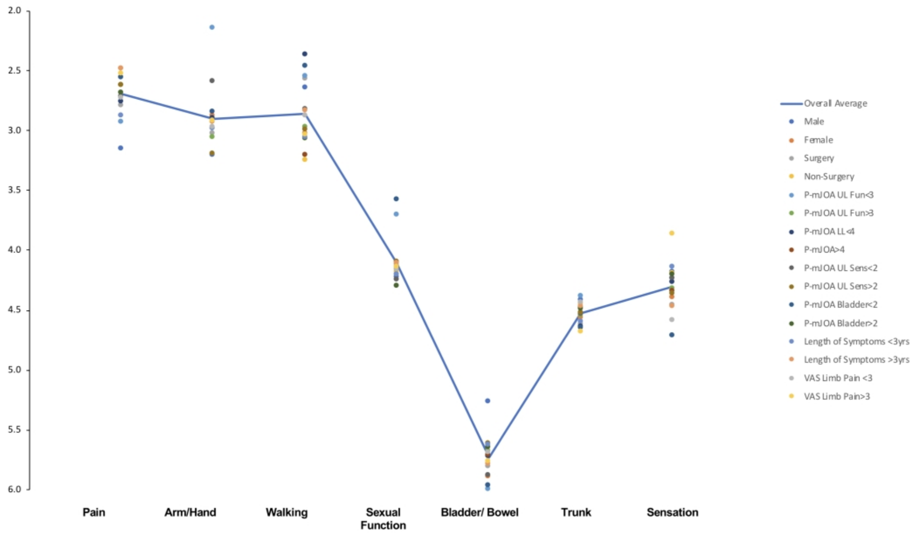

Figure 3: Impact of baseline characteristics on recovery priority average rankings. The scatter plot represents the mean ranking for each subgroup investigated. The black line represents the overall average. Despite some discrepancies between subgroups, pain, arm/hand and walking function were consistently the top three priorities for patients. Bladder/bowel function was not a recovery priority.

Supplementary Data 1: Results from the missing data analysis. Patients completing the survey in full, were more likely to have undergone surgery than those who did not (*p* = 0.04). Data is presented as mean +/- standard deviation, unless specified as a percentage. Numbers within brackets indicate data points for the respective variable with incomplete data.

	INCOMPLETE SURVEYS (N<178)	COMPLETED SURVEYS (N=481)	P
**%MALE**	16.3% (7/43)	28.9%	.076
**AGE**	55.1 +/-10.9 (43)	53.6 +/- 9.9	.344
**%SURGERY**	35.9% (46/128)	46.0%	.043
**LENGTH OF SYMPTOMS (YRS)**	19% (128)	73%	.304
**0-1**	18.0% (23)	15.0% (72)	
**1-3**	21.9% (28)	29.1% (140)	
**3-10**	35.2% (45)	37.6% (181)	
**10-25**	18.8% (24)	15.4% (74)	
**25+**	6.3% (8)	2.9% (14)	
**LIMB PAIN VAS**	3.6 +/- 1.9 (17)	3.7 +/- 2.5	.854
**MJOA**	12.2 +/- 3.2 (47)	11.9 +/- 3.0	.364

For peer review only

Supplementary Data 2: Summary of group differences between investigated variables. Highlighted cells represent significant differences in the proportion of respondents per group. Further analysis revealed that differences followed a logical course: patients who had had symptoms for longer or undergone surgery were more likely to have severe disease. In addition, patients with more severe disease were likely to have higher limb pain scores.

	N	Age (±SD)		Male (%)		Undergone Surgery (%)		Length of Symptoms <3yrs (%)		mJOA Upper Limb Function <3		mJOA Lower Limb Function <4		mJOA Upper Limb Sensation <2		mJOA Bladder Function <2		Mean VAS Limb Pain <3	
Gender, Male	140	55.8	10.3			64	46%	71	51%	20	14%	51	36%	50	36%	30	21%	88	63%
Gemder, Female	341	52.8	9.5			157	46%	141	41%	38	11%	107	31%	143	42%	75	22%	205	60%
Undergone Surgery	221	53.1	8.7	64	29%			83	38%	31	14%	90	41%	101	46%	53	24%	135	61%
Not Undergone Surgery	260	54	10.7	74	28%			129	50%	27	10%	68	26%	92	35%	53	20%	158	61%
mJOA Upper Limb Function <3	58	56.7	9.3	19	33%	31	53%	13	22%			46	79%	52	90%	29	50%	21	36%
mJOA Upper Limb Function >3	423	53.2	9.8	119	28%	190	45%	199	47%			112	26%	141	33%	76	18%	272	64%
mJOA Lower Limb Function <4	158	55.4	10.1	50	32%	90	57%	51	32%	46	29%			91	58%	61	39%	85	54%
mJOA Lower Limb Function >4	323	52.7	9.5	88	27%	131	41%	161	50%	12	4%			102	32%	44	14%	208	64%
mJOA Upper Limb Sensation <2	193	53.7	9.1	48	25%	101	52%	78	40%	52	27%	91	47%			67	35%	86	45%
mJOA Upper Limb Sensation >2	288	53.3	10.3	90	31%	120	42%	134	47%	6	2%	67	23%			38	13%	207	72%
mJOA Bladder Function <2	105	54	9.6	29	28%	53	50%	40	38%	29	28%	61	58%	67	64%			58	55%
mJOA Bladder Function >2	376	53.5	9.9	109	29%	168	45%	172	46%	29	8%	97	26%	126	34%			235	63%
Length of Symptoms <3 years	212	52.8	10.8	71	33%	83	39%			13	6%	51	24%	78	37%	40	19%	136	64%
Length of Symptoms >3 years	269	54.2	8.9	67	25%	138	51%			45	17%	107	40%	115	43%	65	24%	157	58%
Best Limb Pain VAS <3	293	53.3	11	88	30%	135	46%	136	46%	21	7%	85	29%	86	29%	58	20%		
Best Limb Pain VAS >3	188	53.6	8.6	52	28%	86	46%	76	40%	37	20%	73	39%	107	57%	47	25%		

For peer review only

Supplementary Data 3: Mean ranking for recovery domains for baseline characteristics. Based on average rankings, the top ranked domain is highlighted. Whilst pain, walking and arm/hand function remained the priorities, for respondents who were male or had undergone surgery, or had impaired upper, lower or bladder function, arm/hand function had the top ranking. For patients with impaired upper limb sensation, walking function was the priority.

	Pain (SD)		Walking (SD)		Arm/Hand Function (SD)		Sexual Function (SD)		Bladder/ Bowel (SD)		Trunk Function (SD)		Sensation (SD)	
Gender, Male	3.1	(2.2)	3.2	(1.6)	2.6	(1.6)	4.2	(1.8)	5.3	(1.7)	4.4	(1.6)	4.2	(2.3)
Gender, Female	2.5	(2.0)	2.9	(1.4)	3.0	(1.7)	4.1	(1.6)	5.9	(1.6)	4.6	(1.5)	4.4	(2.0)
Undergone Surgery	2.8	(2.1)	3.0	(1.4)	2.6	(1.6)	4.1	(1.6)	5.8	(1.6)	4.6	(1.6)	4.5	(2.0)
Not Undergone Surgery	2.6	(2.0)	2.9	(1.5)	3.2	(1.7)	4.2	(1.7)	5.6	(1.7)	4.4	(1.5)	4.2	(2.1)
mJOA Upper Limb Function <3	2.9	(2.0)	2.1	(1.3)	2.5	(1.3)	3.7	(1.6)	6.0	(1.7)	4.4	(1.3)	4.2	(2.1)
mJOA Upper Limb Function >3	2.6	(2.0)	3.1	(1.5)	3.0	(1.7)	4.2	(1.7)	5.7	(1.7)	4.5	(1.6)	4.3	(2.1)
mJOA Lower Limb Function <4	2.8	(2.0)	2.9	(1.5)	2.4	(1.5)	4.1	(1.6)	5.7	(1.7)	4.6	(1.4)	4.3	(2.1)
mJOA Lower Limb Function >4	2.6	(2.1)	3.0	(1.5)	3.2	(1.7)	4.2	(1.7)	5.7	(1.7)	4.5	(1.6)	4.3	(2.0)
mJOA Upper Limb Sensation <2	2.7	(2.1)	2.6	(1.3)		(1.5)	4.2	(1.5)	5.9	(1.6)	4.5	(1.5)	4.2	(2.1)
mJOA Upper Limb Sensation >2	2.6	(2.0)	3.2	(1.6)	3.0	(1.8)	4.1	(1.8)	5.6	(1.7)	4.5	(1.6)	4.4	(2.1)
mJOA Bladder Function <2	2.6	(1.8)	2.8	(1.6)	2.5	(1.4)	3.6	(1.4)	6.0	(1.3)	4.7	(1.6)	4.7	(2.0)
mJOA Bladder Function >2	2.7	(2.1)	3.0	(1.5)	3.1	(1.7)	4.3	(1.7)	5.6	(1.7)	4.5	(1.5)	4.2	(2.1)
Length of Symptoms <3 years	2.9	(2.2)	3.0	(1.5)	3.1	(1.6)	4.2	(1.7)	5.6	(1.7)	4.6	(1.5)	4.1	(2.1)
Length of Symptoms >3 years	2.5	(1.9)	2.9	(1.4)	2.8	(1.7)	4.1	(1.7)	5.8	(1.6)	4.5	(1.5)	4.5	(2.1)
Best Limb Pain VAS <3	2.7	(2.1)	3.0	(1.5)	2.9	(1.7)	4.2	(1.7)	5.7	(1.7)	4.4	(1.5)	4.6	(2.0)
Best Limb Pain VAS >3	2.5	(2.0)	2.9	(1.5)	3.0	(1.6)	4.1	(1.6)	5.8	(1.6)	4.7	(1.6)	3.9	(2.2)

For peer review only

Supplementary Data 4: Questionnaire. The questions relevant to this study were developed, piloted and embedded within an ongoing initiative investigating patient reporting of DCM. The questions pertaining to the data points required for this study are presented, including their question number and options for selection. Question 35 about age was the only question where respondents were asked to type in a specific answer. The answer format was electronically validated to require an integer, prompting users to specify to the nearest year.

Questions generating data points required for this study	
5	How long have you suffered with cervical myelopathy?<br>0-1 year<br>1-3 years<br>3-10 years<br>10-25 years<br>>25 years
7	Have you undergone surgery for cervical myelopathy?<br>Yes<br>No
23	Currently, please indicate the intensity of the current, best and worst pain affecting your arms or legs over the past 24h on a scale of 0 (no pain) to 10 (worst pain imaginable)<br>0<br>1<br>2<br>3<br>4<br>5<br>6<br>7<br>8<br>9<br>10
29	How does cervical myelopathy affect your arms and hands? *Choose the statement that best fits: I am*<br>- Unable to move my hands<br>- Unable to eat with a spoon but am able to move my hands<br>- Unable to button my shirt but able to eat with a spoon<br>- Able to button my shirt with great difficulty<br>- Able to button my shirt with slight difficulty<br>- Not having any trouble using my hands.

For peer review only

30	How does cervical myelopathy affect your legs? *Choose the statement that best fits: I am*<br><br>- Completely unable to move legs at all and have no feeling in legs<br>- Having feeling in legs but not able to move them at all<br>- Able to move my legs but am unable to walk<br>- Able to walk on flat floor with a walking aid (cane or crutch)<br>- Able to walk up and/or downstairs with the aid of a handrail<br>- Able to walk up and/or downstairs without handrail but I notice moderate-to-significant lack of stability/feeling of imbalance when I walk<br>- Able to walk unaided (no crutches, canes, walker) with smooth reciprocation (ie, legs move smoothly) but I still notice mild lack of stability/feeling of imbalance when walking<br>- Able to walk without any problems of imbalance or instability
31	How does cervical myelopathy affect your arms and hands? *Choose the statement that best fits: I have*<br><br>- Complete loss of feeling in hands<br><br>- Severe loss of feeling, or have pain in my hands<br><br>- Mild loss of feeling in hands<br><br>- No loss of feeling in hands
32	How does cervical myelopathy affect your bladder? *Choose the statement that best fits: I am*<br><br>- Am completely unable to control urination<br><br>- Have marked difficulty controlling urination<br><br>- Have mild to moderate difficulty controlling urination<br><br>- No difficulty controlling urination
33	Effective medical research should target the needs of patients. The consequences of spinal cord injury can be classified into 7 different

For peer review only

	categories. In DCM, the patient priorities are not known.<br><br>For you as a patient, what are the research priorities for you (please rank where 1 is the most important and 7 is the least important)? What would you like researchers to focus on?<br><br>-Elimination of Pain<br>-Arm/Hand Function<br>-Walking Function<br>-Bladder/Bowel Function<br>-Sexual Function<br>-Upper Body/Trunk Function<br>-Normal Sensation
34	Are you male or female?<br><br>Male<br>Female
35	How old are you?

For peer review only

STROBE Statement

Checklist of items that should be included in reports of observational studies

Section/Topic	Item No	Recommendation	Reported on Page No
**Title and abstract**	1	(*a*) Indicate the study's design with a commonly used term in the title or the abstract	2
		(*b*) Provide in the abstract an informative and balanced summary of what was done and what was found	2
**Introduction**			
Background/rationale	2	Explain the scientific background and rationale for the investigation being reported	4-5
Objectives	3	State specific objectives, including any prespecified hypotheses	5
**Methods**			
Study design	4	Present key elements of study design early in the paper	6-7
Setting	5	Describe the setting, locations, and relevant dates, including periods of recruitment, exposure, follow-up, and data collection	6-7
Participants	6	(*a*) *Cohort study*—Give the eligibility criteria, and the sources and methods of selection of participants. Describe methods of follow-up<br>*Case-control study*—Give the eligibility criteria, and the sources and methods of case ascertainment and control selection. Give the rationale for the choice of cases and controls<br>*Cross-sectional study*—Give the eligibility criteria, and the sources and methods of selection of participants	6-7
		(*b*) *Cohort study*—For matched studies, give matching criteria and number of exposed and unexposed<br>*Case-control study*—For matched studies, give matching criteria and the number of controls per case	
Variables	7	Clearly define all outcomes, exposures, predictors, potential confounders, and effect modifiers. Give diagnostic criteria, if applicable	6-7
Data sources/measurement	8*	For each variable of interest, give sources of data and details of methods of assessment (measurement). Describe comparability of assessment methods if there is more than one group	6-7
Bias	9	Describe any efforts to address potential sources of bias	6-7
Study size	10	Explain how the study size was arrived at	6-7
Quantitative variables	11	Explain how quantitative variables were handled in the analyses. If applicable, describe which groupings were chosen and why	6-7
Statistical methods	12	(*a*) Describe all statistical methods, including those used to control for confounding	7
		(*b*) Describe any methods used to examine subgroups and interactions	7
		(*c*) Explain how missing data were addressed	7
		(*d*) *Cohort study*—If applicable, explain how loss to follow-up was addressed<br>*Case-control study*—If applicable, explain how matching of cases and controls was addressed<br>*Cross-sectional study*—If applicable, describe analytical methods taking account of sampling strategy	7
		(*e*) Describe any sensitivity analyses	

For peer review only - http://bmjopen.bmj.com/site/about/guidelines.xhtml

Section/Topic	Item No	Recommendation	Reported on Page No
**Results**			
Participants	13*	(a) Report numbers of individuals at each stage of study—eg numbers potentially eligible, examined for eligibility, confirmed eligible, included in the study, completing follow-up, and analysed	8
		(b) Give reasons for non-participation at each stage	N/A
		(c) Consider use of a flow diagram	N/A
Descriptive data	14*	(a) Give characteristics of study participants (eg demographic, clinical, social) and information on exposures and potential confounders	8
		(b) Indicate number of participants with missing data for each variable of interest	Supplementary Data 2
		(c) *Cohort study*—Summarise follow-up time (eg, average and total amount)	N/A
Outcome data	15*	*Cohort study*—Report numbers of outcome events or summary measures over time	N/A
		Case-control study—Report numbers in each exposure category, or summary measures of exposure	N/A
		Cross-sectional study—Report numbers of outcome events or summary measures	12-13
Main results	16	(*a*) Give unadjusted estimates and, if applicable, confounder-adjusted estimates and their precision (eg, 95% confidence interval). Make clear which confounders were adjusted for and why they were included	8
		(*b*) Report category boundaries when continuous variables were categorized	Supplementary Data 1,3 & 4
		(*c*) If relevant, consider translating estimates of relative risk into absolute risk for a meaningful time period	N/A
Other analyses	17	Report other analyses done—eg analyses of subgroups and interactions, and sensitivity analyses	8-9
**Discussion**			
Key results	18	Summarise key results with reference to study objectives	10
Limitations	19	Discuss limitations of the study, taking into account sources of potential bias or imprecision. Discuss both direction and magnitude of any potential bias	11
Interpretation	20	Give a cautious overall interpretation of results considering objectives, limitations, multiplicity of analyses, results from similar studies, and other relevant evidence	10-11
Generalisability	21	Discuss the generalisability (external validity) of the study results	10-11
**Other Information**			
Funding	22	Give the source of funding and the role of the funders for the present study and, if applicable, for the original study on which the present article is based	1

**Give information separately for cases and controls in case-control studies and, if applicable, for exposed and unexposed groups in cohort and cross-sectional studies.*

For peer review only - http://bmjopen.bmj.com/site/about/guidelines.xhtml

Note: An Explanation and Elaboration article discusses each checklist item and gives methodological background and published examples of transparent reporting. The STROBE checklist is best used in conjunction with this article (freely available on the Web sites of PLoS Medicine at http://www.plosmedicine.org/, Annals of Internal Medicine at http://www.annals.org/, and Epidemiology at http://www.epidem.com/). Information on the STROBE Initiative is available at www.strobe-statement.org.

For peer review only

For peer review only - http://bmjopen.bmj.com/site/about/guidelines.xhtml
